# Sigmatropic rearrangement enables access to a highly stable spirocyclic nitroxide for protein spin labelling†

**DOI:** 10.1039/d5cc00472a

**Published:** 2025-03-27

**Authors:** Mateusz P. Sowiński, Elena M. Mocanu, Hannah Ruskin-Dodd, Aidan P. McKay, David B. Cordes, Janet E. Lovett, Marius Haugland-Grange

**Affiliations:** a Department of Chemistry, UiT The Arctic University of Norway 9037 Tromsø Norway marius.haugland-grange@uit.no; b SUPA School of Physics and Astronomy and BSRC, University of St Andrews North Haugh St Andrews KY16 9SS UK; c EaStCHEM School of Chemistry, University of St Andrews North Haugh St Andrews KY16 9ST UK

## Abstract

Spin labelling enables the study of biomolecules using electron paramagnetic resonance (EPR) spectroscopy. Here, we describe the synthesis of a cysteine-reactive spin label based on a spirocyclic pyrrolidinyl nitroxide containing an iodoacetamide moiety. The spin label was shown to be highly persistent under reducing conditions while maintaining excellent EPR relaxation parameters up to a temperature of 180 K. After successful double spin labelling of a calmodulin variant, interspin distances were measured by the EPR experiment double electron–electron resonance (DEER) at 120 K.

## Abstract 2

An Overman rearrangement gives access to a spirocyclic nitroxide spin label that is resistant to degradation and enables DEER measurements at liquid nitrogen temperatures.

## Graphical abstract

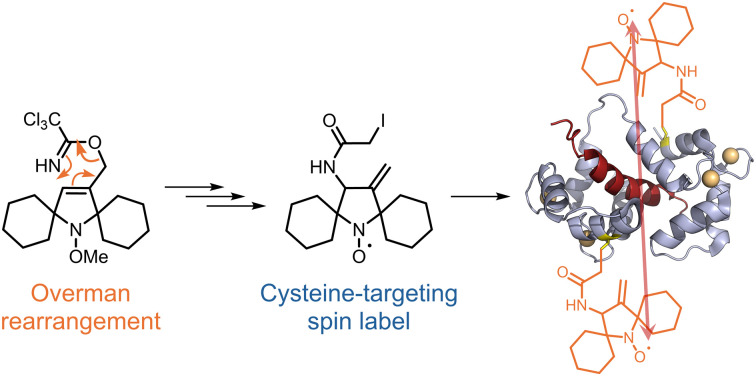

Electron paramagnetic resonance (EPR) spectroscopy has emerged as an important technique for examining the structures and dynamics of biomolecules. The considerable applicability of this tool is attributed to its ability to function under biological conditions, independently of the size of the biomolecules, with high accuracy and sensitivity in detecting even small changes.^1–4^ EPR techniques typically require the presence of paramagnetic centres, which are not commonly found in predominantly diamagnetic biopolymers. Therefore, paramagnetic probes can be introduced into specific regions of the target molecule of interest, a strategy termed site-directed spin labelling (SDSL).^5^ Nitroxide-based probes have gained a unique position in this field, among other factors due to their small size and minimal impact on the biomolecular structure.^6,7^ In order for a nitroxide to be suitable and widely applicable as a spin label, particularly in an intracellular environment, it must have sufficient aqueous solubility, efficient labelling reactivity (usually with the sulfhydryl group of a cysteine amino acid), be minimally perturbing to the protein structure, be resilient to reducing conditions, and have sufficiently long relaxation times (particularly for transverse relaxation, *i.e.* the phase memory time *T*_m_).^6^ These properties are influenced by structural factors such as ring size and saturation, the α-substituents, or electronic effects of functional groups.^7^ Saturated five-membered pyrrolidinyl nitroxides have greater kinetic stability towards reducing agents than their unsaturated pyrrolinyl or six-membered piperidinyl counterparts.^8–10^ Increasing the bulk of the α-substituents, meanwhile, can be beneficial to reduction stability, but also detrimental for the relaxation parameters. For example, tetraethyl substituents can provide exceptional stability compared to tetramethyl substitution;^8,9,11,12^ however, as rotation of the CH_3_ of the ethyl groups leads to poor relaxation times at temperatures above 70 K, measurements require costly cryogenic conditions using liquid helium.^13^ Optimal relaxation parameters are shown by conformationally rigid nitroxides featuring spirocycles at the α-positions, which notably enable measurements at elevated temperatures.^13^

Our previous research revealed that the conformations adopted by these spirocyclic scaffolds is another important factor influencing nitroxide stability.^14^ Recently, we have also developed nitroxides based on an *exo*-methylene substituted spirocyclic scaffold and extensively analysed their properties.^15^ In this work, we build on these prior discoveries to develop a novel spin label based on an *exo*-methylene substituted spirocyclic nitroxide, and implement it in the examination of proteins through double electron–electron resonance (DEER; also known as pulsed electron double resonance, PELDOR) experiments.^3,4^ This new spin label benefits from a minimum number of rotatable bonds linking the nitroxide core to the protein, maintains high stability also when attached to a protein scaffold, and enables DEER measurements up to 180 K.

The synthesis of the new spin label began by the introduction of the spirocyclohexyl substituents by ketone exchange with 1,2,2,6,6-pentamethylpiperidin-4-one 1 and cyclohexanone, resulting in the spirocyclic piperidone 2 (Scheme 1).^16^ Bromination led to the quantitative formation of hydrobromide salt 3, which was then subjected to a Favorskii rearrangement^17^ to form spirocyclic pyrroline ester 4 in good yield. Oxidation of secondary amine 4 with *m*-chloroperbenzoic acid (*m*-CPBA) gave the nitroxide 5. As the next transformations required harsh conditions, the nitroxide moiety was at this point protected as the methoxyamine 6 using previously reported Fenton chemistry.^18^ The ester group in 6 was reduced with diisobutylaluminium hydride (DIBAL-H) to the alcohol 7 in very good yield. This allylic alcohol was then subjected to a two-step Overman rearrangement as the key step in our synthetic strategy. Alcohol 7 was first treated with trichloroacetonitrile in the presence of a base, yielding a trichloroacetimidate. This labile intermediate was immediately subjected to a base-catalysed, microwave-assisted [3,3]-sigmatropic rearrangement to form the *exo*-methylene substituted trichloroacetamide 8 in excellent yield over two steps. Deprotection of the methoxyamine by treatment with *m*-CPBA resulted in a Cope-type elimination to the nitroxide 9,^18^ albeit in moderate yield due to competing epoxidation of the double bond. The crystalline trichloroacetamide 9 was analysed using X-ray crystallography, which revealed that the nitroxide adopts a semi-open conformation of the spirocyclohexyl moieties in the solid state (Scheme 1). Previous reports suggest that such conformations may limit the accessibility of potential reducing agents to the nitroxide radical centre, thus enhancing its kinetic stability.^19^ Base-promoted hydrolysis of the trichloroacetamide moiety provided primary amine 10 in excellent yield, which was thereafter transformed to chloroacetamide 11 by acylation. Finally, a Finkelstein reaction provided the *exo*-methylene substituted spirocyclic pyrrolidinyl iodoacetamide 12, which was further used for protein spin labelling.

**Scheme 1:**
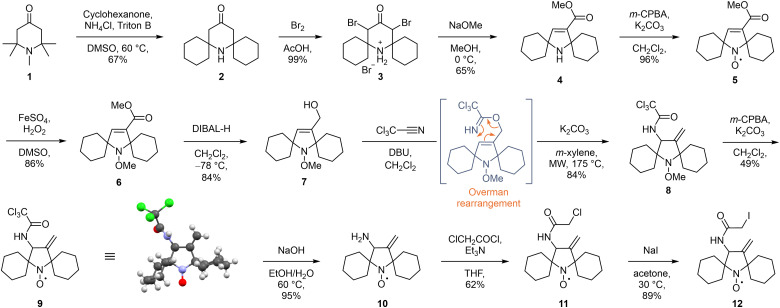
Synthetic route towards *exo*-methylene substituted spirocyclic pyrrolidinyl nitroxide spin label 12 and X-ray structure of the nitroxide 9.

Calmodulin (CaM) is a relatively small protein abundant in eukaryotic cells that plays a pivotal role in sensing and responding to modulation of calcium levels by regulating the activity of a large number of proteins important to human health.^20^ As a biologically important, thoroughly studied and structurally well-understood protein, we identified CaM as a suitable protein to benchmark the use of iodoacetamide 12 for spin labelling. More specifically, we chose to employ the complex formed by Ca^2+^-bound holo-CaM and the synthetic peptide M13 *via* a GG linker (CaM/M13), which mimics the CaM-binding domain of rabbit skeletal muscle myosin light chain kinase, as our model protein (Fig. 1).^21^ As the native CaM/M13 amino acid sequence lacks cysteine residues suitable for labelling with iodoacetamide spin labels, a double mutant with cysteines at residues 34 and 146 (highlighted yellow residues in Fig. 1)^22^ was produced by mutagenesis (see the ESI† for details).^23^

**Fig. 1:**
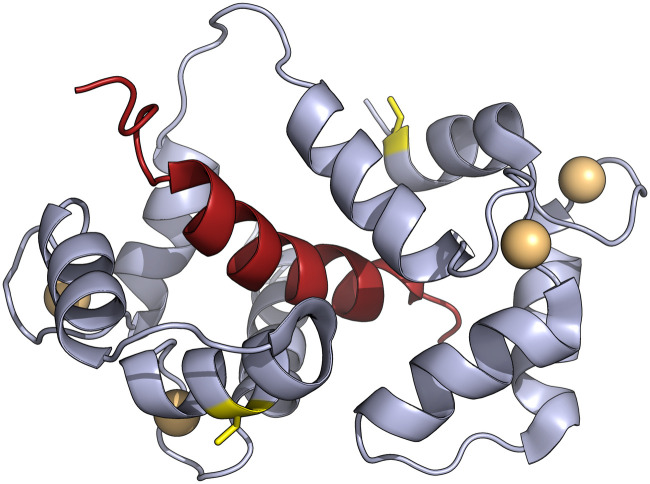
Double cysteine variant of holo-calmodulin (lilac) complexed with the peptide M13 (red). The two cysteines at positions 34 and 146 are highlighted in yellow, and Ca^2+^-ions are shown as orange spheres. Based on PDB ID 2BBM (see the ESI† for details).^22^

Spin labelling of the double cysteine variant of CaM/M13 was performed using 50 μM protein and 500 μM nitroxide 12 in HEPES buffer (40 mM, pH 7.5) and NaCl solution (150 mM). Due to the light sensitive nature of iodoacetamides, the mixture was incubated in darkness at 4 °C for 16 h. This protocol yielded a 3 : 10 ratio of singly- and doubly-labelled protein, with a notable 77% efficiency in double labelling according to analysis by ESI-MS (see the ESI,† Fig. S1). The doubly-labelled protein was subsequently isolated after functionalising any unreacted free cysteines with biotin and purification using streptavidin-coated beads (see the ESI,† Fig. S2).^23^ Spirocyclic pyrrolinyl and pyrrolidinyl nitroxide spin labels have been synthesized previously, but to the best of our knowledge, this is the first example of five-membered nitroxides with spirocyclohexyl moieties being used in protein spin labelling.^10,19,24^

To assess the stability of the spin label in a reducing environment, we recorded X-band continuous wave (CW) EPR spectra of the doubly-labelled protein (250 μM in HEPES buffer, pH 7.5) in the presence of a 32-fold (16-fold per label) excess of the reducing agent sodium ascorbate (Fig. 2). The data shows a 50% decrease in the intensity of the CW spectrum over approximately 20 minutes, and a 66% decrease after 40 minutes. These measurements confirm that the EPR signal of spin label 12 when attached to the protein remains sufficiently strong for long enough to permit spectroscopic measurements, possibly even in challenging reducing media such as the intracellular environment.

**Fig. 2:**
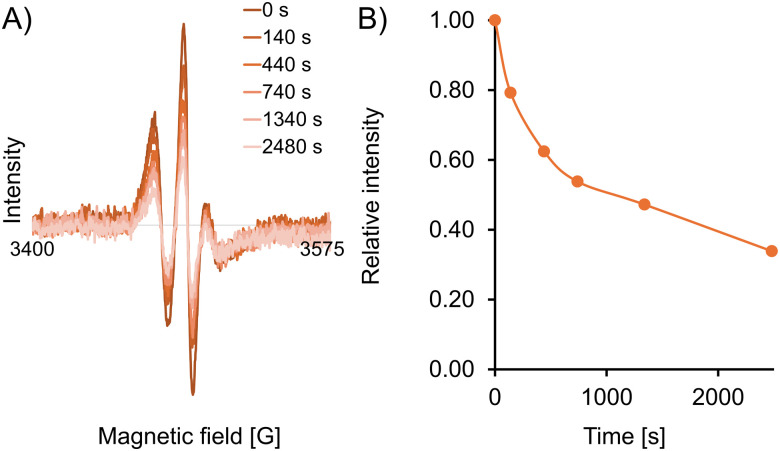
Reduction stability of labelled protein. (A) Superimposed CW EPR spectra of CaM/M13 doubly labelled with 12 (250 μM) with sodium ascorbate (32 equiv.) in HEPES buffer at 295 K. (B) Intensity decay of the low-field line of the CW EPR spectrum over time.

Next, the relaxation profile of the new spin label was investigated by determining the phase memory time *T*_m_ for the spin label 12 in a protonated medium, and the doubly-labelled protein in a solution containing deuterated solvents, by pulsed EPR spectroscopy (Fig. 3). Glycerol (50%) was added to ensure glass formation upon freezing, although this might affect the protein and absolute *T*_m_ values. *T*_m_ reflects the intensity decay of an electron spin echo during pulsed EPR experiments, and this factor contributes significantly to the resolution and accuracy of DEER distance measurements. Spin echo decay curves were recorded at Q-band at various temperatures (Fig. 3A and B), and numerical values for *T*_m_ were extracted (see the ESI†). Spin label 12 has a *T*_m_ of 4.26 μs at 50 K, which only significantly decreases from 160 K (*T*_m,160K_ = 3.28 μs, Fig. 3A). *T*_m_ at 180 K (just above the glass melting temperature) is 2.35 μs, which corresponds to a 45% decay and is in agreement with our previous results performed on a similar nitroxide scaffold.^15^ The same pattern is evident for the labelled protein, but with phase memory times of approximately double duration (8.56 μs at 50 K, Fig. 3B) due to the protein being measured in a deuterated solvent mixture. A comparison of the logarithm of the phase memory decay rates (*i.e.* the reciprocal of *T*_m_) across temperatures is shown in Fig. 3C. It is evident that the absence of rotatable methyl groups and relative rigidity of the nitroxide scaffold 12 results in excellent relaxation behaviour both in the free label and when attached to the protein. These findings validate the utility of spin label 12 for the investigation of proteins at elevated temperatures.

**Fig. 3:**
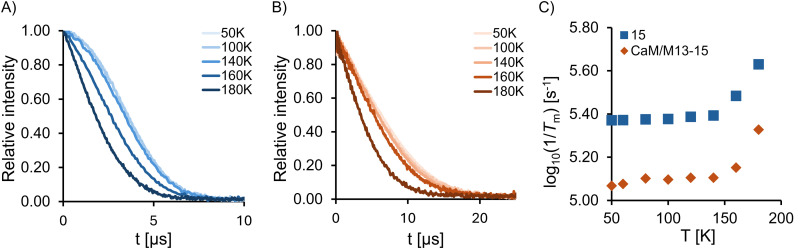
Spin echo decay curves for determining *T*_m_ measured by 2-pulse Q-band EPR spectroscopy. The time corresponds to twice the incremented time from the first data point. (A) Spin echo decay curves for the nitroxide 12 (50 μM solution in glycerol/PBS buffer/DMSO 1000 : 999: 1 v/v) at various temperatures. The curves at 50 K and 100 K are fully overlapping. (B) Spin echo decay curves for the CaM/M13 protein doubly labelled with 12 (80 μM solution in 40 mM HEPES buffer, pH 7.5, 150 mM NaCl, 10 mM CaCl_2_, D_2_O, then 50% v/v glycerol-d_8_) at various temperatures. (C) Logarithm of the reciprocal of *T*_m_*vs.* temperature for nitroxide 12 and doubly-labelled CaM/M13, measured by spin echo decay, at various temperatures. The generally longer *T*_m_ of 12@CaM/M13 is due to the use of deuterated solvent.

DEER experiments were conducted to determine the distance between the spin labels in the doubly-labelled CaM/M13 complex.^3,4,25–27^ The presented data was acquired at 120 K, with distance distributions obtained through analysis with DEERNet (Fig. 4).^28,29^ Additional DEER experiments (at 50 K and 180 K, and analysis with DeerAnalysis) are available in the ESI.†^30^ The bimodal distribution in Fig. 4B has maxima at 36 Å and 42 Å. The intense 36 Å peak coincides well with measurements of this protein using MTSL, a cysteine-selective, five-membered, tetramethyl-substituted spin label. The 42 Å peak matches a simulated distance (chiLife; see ESI,† Fig. S13 for overlay) of the MTSL-labelled protein.^31,32^ This discrepancy implies some protein-label interaction not accounted for by the simulation, which may be partially present also in the larger label 12 that exhibits both distances. We have observed the longer distance for CaM/M13 with even larger and more rigid Gd(iii) spin labels,^23^ where the interaction therefore appears to be absent. Therefore, we conclude that label 12 is able to reveal local minima in the protein-spin label conformational space.

**Fig. 4:**
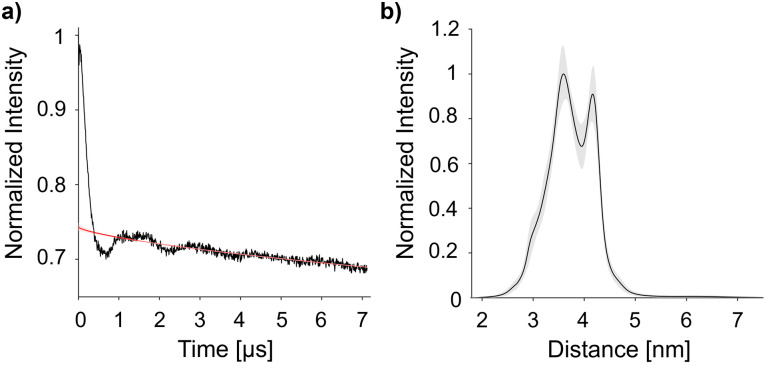
Double electron–electron resonance (DEER) measurement at 120 K. (A) DEER time trace for CaM/M13 doubly labelled with nitroxide 12 with the background fit calculated by DEERNet shown as a red line. (B) The corresponding distance distribution, provided by DEERNet (with 95% confidence represented by the trace shade).^28,29^

In conclusion, we have synthesised an *exo*-methylene substituted pyrrolidinyl nitroxide iodoacetamide spin label with spirocyclohexyl substituents surrounding the paramagnetic centre. This label was successfully attached to a double mutant of calmodulin bound to the M13 peptide with high labelling efficiency. The protein-appended nitroxide showed high stability in the presence of excess ascorbate as a model system for a reducing environment. The lack of methyl group rotation resulted in long relaxation times up to 180 K, which enabled DEER measurements of interspin distance distributions even at elevated temperatures. These results provide a promising basis for the future application of spirocyclic pyrrolidinyl nitroxide spin labels in intracellular EPR measurements.

## Data availability

A previous version of this work prior to peer review has been posted on a preprint server.^33^ Crystallographic data for 9 has been deposited at the CCDC under deposition number 2406207 and can be obtained from https://www.ccdc.cam.ac.uk/structures. Additional data for this article is available at DataverseNO at https://doi.org/10.18710/VC1H83.^34^

## Conflicts of interest

There are no conflicts to declare.

## Supplementary Material

CC-061-D5CC00472A-s001

CC-061-D5CC00472A-s002
