# Supplementary material for: Sigmatropic rearrangement enables access to a highly stable spirocyclic nitroxide for protein spin labelling
Source: Chem Commun (Camb). 2025 Mar 27;61(37):6755–8. doi: 10.1039/d5cc00472a (PMC11973475; doi:10.1039/d5cc00472a)
Supplement: CC-061-D5CC00472A-s001 [file CC-061-D5CC00472A-s001.pdf]

Supplementary Information (SI) for ChemComm.
This journal is © The Royal Society of Chemistry 2025

Supplementary Information

Sigmatropic rearrangement enables access to a highly stable spirocyclic nitroxide for protein spin labelling

Mateusz P. Sowiński,[a] Elena M. Mocanu,[b] Hannah Ruskin-Dodd,[b] Aidan P. McKay,[c] David B. Cordes,[c] Janet E. Lovett,[b] and Marius Haugland-Grange*[a]

* *e-mail: marius.haugland-grange@uit.no*

[a] Department of Chemistry, UiT The Arctic University of Norway, 9037 Tromsø, Norway

[b] SUPA School of Physics and Astronomy and BSRC, University of St Andrews, North Haugh, St Andrews KY16 9SS, UK

[c] EaStCHEM School of Chemistry, University of St Andrews, North Haugh, St Andrews KY16 9ST, UK

Contents

1. General procedures and materials 3
2. Synthesis and characterization of nitroxides and intermediates 4
3. Crystallography data 14
4. Preparation of spin-labelled protein 15
   4.1. Preparation of CaM/M13 34C 146C 15
   4.2. Visualisation of CaM/M13 34C 146C 15
   4.3. Spin labelling 15
5. EPR spectroscopy 18
   5.1. CW spectra of nitroxides and labelled protein 18
   5.2. Kinetic studies 21
   5.3. Relaxation measurements 21
   5.4. DEER experiment 23

NMR spectra 27

References 37

1. General procedures and materials

All reactions sensitive to moisture or air were performed under inert conditions using Schlenk techniques. All reagents, obtained from Acros, Alfa, Sigma-Aldrich, TCI, and VWR were used directly as supplied unless otherwise noted. Phosphate-buffered saline (PBS) (10 ×, pH 7.4, Gibco™ product line, 1551.72 mM NaCl, 29.66 mM Na_2HPO_4, and 10.58 mM KH_2PO_4) was purchased from Thermo Fisher Scientific. Anhydrous solvents were dried by pre-storing over activated 3 or 4 Å molecular sieves under argon. Thin layer chromatography (TLC) was used to monitor reaction progress. TLC analysis was performed on pre-coated aluminium-based plates (TLC Silica gel 60 F_{254}, Supelco) and plates were developed under UV irradiation (254 nm) or with $KMnO_4$ staining and subsequent heating. Column chromatography was performed with silica gel (Silica gel 60, irregular 40–63 µm for flash chromatography, VWR Chemicals). ^{1}H Nuclear magnetic resonance (NMR) spectra were recorded at ambient probe temperatures on a Bruker 9.4 Tesla Avance III HD system equipped with a SmartProbe (broad band) operating at 400 MHz. Spectra were recorded in $CDCl_3$, and referenced to residual protons in $CDCl_3$ (δ 7.26), or benzene (δ 7.36) for nitroxides reduced with phenylhydrazine. Chemical shifts (δ) are reported as parts per million (ppm). Coupling constants (*J*) are given in Hz to an accuracy of 0.1 Hz. The following abbreviations have been used for multiplicity assignments: "s" for singlet, "d" for doublet, "t" for triplet, "td" for triplet of doublets, and "m" for multiplet. Assignment of ^{1}H resonances were determined based on unambiguous chemical shift, coupling patterns, by analysis of 2D NMR (COSY, HSQC and/or HMBC) and/or by analogy to fully interpreted spectra of closely structurally related compounds. ^{13}C NMR spectra (^{1}H decoupled) were recorded in $CDCl_3$, at ambient probe temperatures on the instrument mentioned above, operating at 101 MHz, and referenced to the $CDCl_3$ solvent signal (δ 77.16). High-resolution mass spectra (HRMS) were recorded by direct injection of the compounds as solutions in methanol or acetonitrile on a Thermo Scientific Orbitrap Exploris 120 Mass spectrometer, using a dual electrospray ionization (ESI) probe. IR spectra were obtained on an Agilent Technologies Cary 630 FTIR 318 spectrometer equipped with a ZnSe crystal ATR module. Absorptions are reported in wavenumbers (cm^{-1}).

2. Synthesis and characterization of nitroxides and intermediates

2.1. 1,2,2,6,6-Pentamethylpiperidin-4-one (1)

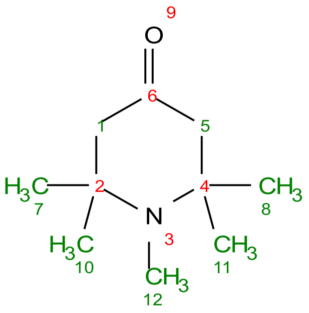

Formic acid (2.3 mL, 61 mmol) was added to a heated solution (90 °C) of 2,2,6,6-tetaramethylpiperidin-4-one (9.93 g, 60.8 mmol) and paraformaldehyde (4.38 g, 91.1 mmol) in toluene (51 mL). The flask was fitted with a Dean-Stark apparatus, and insulated with aluminium foil, and the reaction mixture was refluxed for 16 h to remove water azeotropically at 140 °C. Then the reaction mixture was allowed to cool to room temperature, and NaOH (1.2 g, 30 mmol) was added. After 1 hour of stirring, the NaOH was removed by filtration, and the filtrate was freed of solvent under reduced pressure. The crude product was purified by distillation under reduced pressure to provide the desired *N*-alkylated product (7.44 g, 43.9 mmol, 72%) as a yellow oil.

b.p. 80 °C at 4.8 mbar; **^{1}H NMR** (400 MHz, $CDCl_3$) δ 2.30 (4H, s, H1, H5), 2.26 (3H, s, H12), 1.07 (12H, s, H7, H8, H10, H11); **^{13}C NMR** (101 MHz, $CDCl_3$) δ 210.0, 58.7, 55.7, 28.6, 27.2.

Data are consistent with literature values.[1]

2.2. 7-Azadispiro[5.1.5^8.3^6]hexadecan-15-one (2)

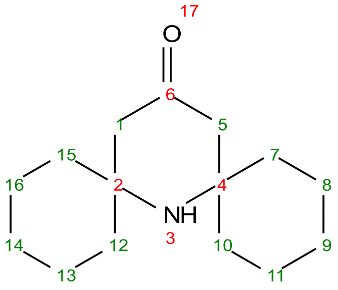

1,2,2,6,6-Pentamethylpiperidin-4-one (**1**, 2.13 g, 12.6 mmol, 1.0 equiv.), NH_4Cl (4.03 g, 75.5 mmol, 6.0 equiv.) and cyclohexanone (3.9 mL, 38 mmol, 3.0 equiv.) were dissolved in DMSO (19 mL) at room temperature and placed under a nitrogen balloon. Triton B (40% in methanol, 2.5 mL) was added dropwise over stirring at room temperature, and the reaction mixture was stirred at 50 °C for 5 h. The mixture was cooled to room temperature, water (20 mL) was added

and the mixture was stirred for a further 30 min. The solution was acidified with conc. HCl (pH < 3) and extracted with diethyl ether (2 × 20 mL). The ether washings were discarded, and the aqueous DMSO solution was adjusted to pH > 9 with NaOH (6 M). The mixture was extracted with ethyl acetate (3 × 20 mL) and the combined extracts were dried over Na_2SO_4. The solvent was removed *in vacuo.* After purification by column chromatography (ethyl acetate / petroleum ether (2:8)), the desired product **2** (1.98 g, 8.41 mmol, 67%) was obtained as white crystals.

R_f 0.38 (ethyl acetate / *n*-pentane (3:7)); **^{1}H NMR** (400 MHz, $CDCl_3$) δ 2.31 (4H, s, H1, H5), 1.70–1.33 (20H, m, H7-16), 1.05 (1H, s, H3); **^{13}C NMR** (101 MHz, $CDCl_3$) δ 211.3, 56.6, 52.2, 40.6, 25.5, 22.2.

Data are consistent with literature values.[2]

2.3. 14,16-Dibromo-15-oxo-7-azadispiro[5.1.5^8.3^6]hexadecan-7-ium bromide (3)

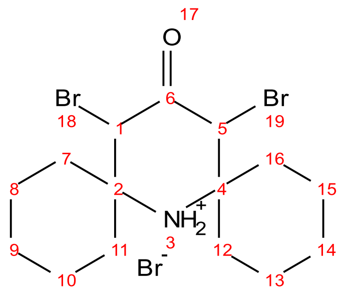

Prepared according to a modified procedure.[2] To a stirred solution of ketone **2** (1.31 g, 5.55 mmol, 1.0 equiv.) in acetic acid (3.0 mL), a solution of Br_2 (1.4 mL, 28 mmol, 5.0 equiv.) in acetic acid (1.8 mL) was added dropwise by glass pipette. (**Caution: bromine is extremely corrosive.**) The solution became deep red initially and an orange precipitate was formed after approximately 30 minutes of stirring. The mixture was stirred at ambient temperature overnight. Diethyl ether (10 mL) was added, and after stirring for 1 minute the precipitate was allowed to settle. The supernatant was carefully removed by glass pipette to a stirring solution of $Na_2S_2O_3$ (aq. sat.). Stirring with diethyl ether and removal of the supernatant after settling was repeated 5 times. The resulting yellow precipitate was dried under high vacuum at ambient temperature to yield the product **3** (2.61 g, 5.50 mmol, 99%) as a yellow powder which was directly used for the next step without further purification.

2.4. Methyl 7-azadispiro[5.1.5^8.2^6]pentadec-14-ene-14-carboxylate (4)

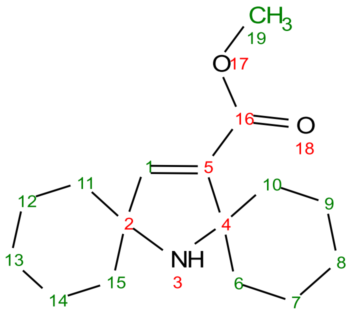

A fresh solution of NaOMe was prepared by dissolving Na (560 mg, 24.4 mmol, 6.0 equiv.) that had been rinsed with heptane, ethanol and again heptane, in dry methanol (8.1 mL) under Ar. (**Caution: Na reacts explosively with water.**) At 0 °C and with stirring, the dried hydrobromide salt **3** (1.92 g, 4.04 mmol, 1.0 equiv.) was added portion-wise to the NaOMe solution. The reaction mixture was stirred at room temperature for 4 h, before the solvent was removed *in vacuo*. The residue was re-dissolved in 10% aqueous Na_2CO_3, then extracted with diethyl ether (3 × 20 mL). The combined ether layers were washed with brine, dried over Na_2SO_4, and concentrated *in vacuo*. The residue was purified by column chromatography (diethyl ether / petroleum ether (0:1 to 15:85)) to afford methyl ester **4** (691 mg, 2.62 mmol, 65%) as a pale-yellow oil.

R_f 0.28 (diethyl ether / *n*-pentane (1:9)); **^{1}H NMR** (400 MHz, $CDCl_3$) δ 6.78 (1H, s, H1), 3.67 (3H, s, H19), 2.03 (2H, td, *J* = 12.7, 5.7 Hz, H10), 1.66–1.02 (20H, m, H6-15); **^{13}C NMR** (101 MHz, $CDCl_3$) δ 164.8, 147.8, 139.3, 67.7, 66.3, 51.1, 39.9, 37.5, 25.5, 25.3, 23.3, 22.5, 22.3.

Data are consistent with literature values.[2]

2.5. Methyl 7-azadispiro[5.1.5^8.2^6]pentadec-14-ene-14-carboxylate-*N*-oxyl (5)

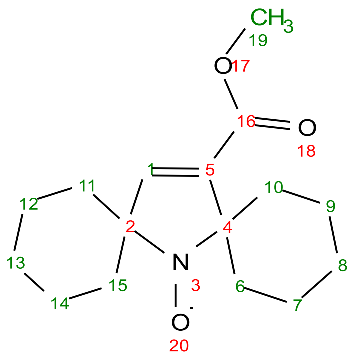

Ester **4** (690 mg, 2.62 mmol, 1.0 equiv.) was dissolved in CH_2Cl_2 (17 mL), and K_2CO_3 (911 mg, 6.59 mmol, 2.5 equiv.) was added at room temperature. The suspension was cooled to 0 °C,

and *m*-CPBA (≤77%; 1.17 g, 5.24 mmol, 2.0 equiv.) was added portion-wise, and the reaction mixture was stirred at room temperature for 16 h. The mixture was washed with 10% aqueous Na_2CO_3 (20 mL), and the aqueous phase was extracted with CH_2Cl_2 (3 × 20 mL). The combined organic phases were dried over Na_2SO_4 and the solvent was removed *in vacuo*. The residue was purified by column chromatography (diethyl ether / *n*-pentane (1:9)) to afford nitroxide **5** (702 mg, 2.52 mmol, 96%) as an orange solid.

Prior to NMR analysis, radical **5** was reduced *in situ* to the corresponding hydroxylamine by addition of phenylhydrazine directly to the NMR tube.

R_f 0.30 (diethyl ether / *n*-pentane (1:9)); **^{1}H NMR** (400 MHz, $CDCl_3$) δ 6.96 (1H, s, H1), 3.73 (3H, s, H19), 2.27–1.04 (20H, m, H6-15); **^{13}C NMR** (101 MHz, $CDCl_3$) δ 164.4, 144.2, 136.9, 72.5, 72.2, 51.1, 35.1, 34.3, 25.4, 24.8, 23.7, 22.9.

Data are consistent with literature values.[2]

2.6. Methyl 7-methoxy-7-azadispiro[5.1.5^8.2^6]pentadec-14-ene-14-carboxylate (6)

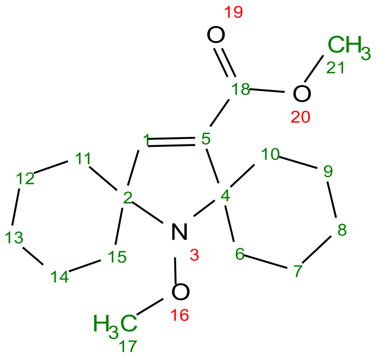

Prepared according to a modified procedure for the preparation of a related compound.[3] Nitroxide **5** (0.479 g, 1.72 mmol, 1.0 equiv.) was dissolved in dimethylsulfoxide (9 mL). To the stirred solution was added $FeSO_4 \cdot 7H_2O$ (1.19 g, 4.30 mmol, 2.5 equiv.). Hydrogen peroxide solution (30% in water; 0.67 mL, 8.6 mmol, 5.0 equiv.) was added dropwise to the stirring solution over 5 minutes. The reaction was stirred for 3 hours at room temperature. After completion, deionised water (10 mL) was added. The aqueous solution was extracted with diethyl ether (3 × 20 mL). The organic phase was dried (Na_2SO_4), filtered and the solvent removed *in vacuo*. The crude mixture was purified by column chromatography (diethyl ether / *n*-pentane (5:95)), providing methoxyamine **6** (0.435 g, 1.48 mmol, 86%) as colourless solid.

R_f 0.68 (diethyl ether / n-*p*entane (5:95)); **IR** (neat, ν_{max} / cm^{-1}) 2926, 2858, 1720, 1636, 1450, 1435, 1303, 1230, 1123, 1050, 912, 773; **^{1}H NMR** (400 MHz, $CDCl_3$) δ 6.98 (1H, s, H1), 3.72 (3H, s, H21), 3.65 (3H, s, H17), 2.24–1.14 (20H, m, H6-15); **^{13}C NMR** (101 MHz, $CDCl_3$) δ 164.8, 144.0, 137.2, 73.5, 72.9, 64.1, 51.4, 41.0, 38.1, 33.4, 31.5, 26.1, 25.0, 24.8, 24.0, 23.6, 22.8; **HRMS** (ESI+) calc. for $C_{17}H_{28}NO_3$ ($[M+H]^+$) 294.2064, found 294.2063.

2.7. (7-Methoxy-7-azadispiro[5.1.5^8.2^6]pentadec-14-en-14-yl)methanol (7)

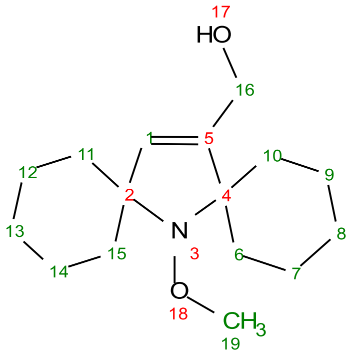

A solution of ester **6** (302 mg, 1.03 mmol, 1.0 equiv.) in dry THF (3.7 mL) under Ar was cooled to –78 °C. A solution of DIBAL (1 M in hexane, 2.6 mL, 2.6 mmol, 2.5 equiv.) was added dropwise, and the reaction mixture was stirred while slowly warming up to –50 °C. After being maintained between –50 °C and –45 °C for 1 h, the reaction was placed in an ice-water bath and diluted with diethyl ether. Then, water (0.1 mL) was added slowly, and the mixture started fuming. A 15% aqueous solution of NaOH (0.1 mL) was added, followed by water (0.25 mL). The mixture was allowed to warm to room temperature and stirred for 30 min. Two spatulas of anhydrous Na_2SO_4 were added, and the mixture was stirred for 30 min and filtered through a cotton plug. The solvent was removed *in vacuo*. After purification by column chromatography (diethyl ether / *n*-pentane (3:7)) the alcohol **7** (229 mg, 0.863 mmol, 84%) was obtained as a colourless oil.

R_f 0.48 (diethyl ether / *n*-pentane (3:7)); **IR** (neat, ν_{max} / cm^{-1}) 3307 (br), 2924, 2852, 1450, 1317, 1204, 1038, 911, 739; **^{1}H NMR** (400 MHz, $CDCl_3$) δ 5.94 (1H, t, J = 1.8 Hz, H1), 4.22 (2H, d, J = 4.3 Hz, H16), 3.64 (3H, s, H19), 1.80–1.08 (20H, m, H6-H15); **^{13}C NMR** (101 MHz, $CDCl_3$) δ 146.4, 126.0, 72.3, 71.7, 64.8, 60.9, 40.6, 38.0, 34.1, 32.7, 26.2, 25.2, 24.9, 23.8; **HRMS** (ESI+) calc. for $C_{16}H_{28}NO_2$ ($[M+H]^+$) 266.2115, found 266.2114.

Analysis of the ^{13}C NMR spectrum was complicated by severe line broadening of several resonances originating from the spirocyclohexyl moieties.

2.8. 2,2,2-Trichloro-*N*-(7-methoxy-15-methylene-7-azadispiro[5.1.5^8.2^6]pentadecan-14-yl)acetamide (8)

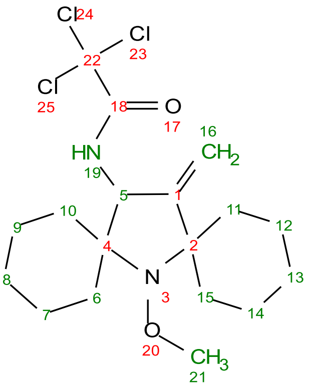

Prepared according to a modified procedure for a related transformation.[4] A solution of alcohol **7** (187 mg, 0.705 mmol, 1.0 equiv.) in dry CH_2Cl_2 (5.6 mL) was gradually treated with DBU (0.260 mL, 1.76 mmol, 2.5 equiv.) and then trichloroacetonitrile (0.110 mL, 1.06 mmol, 1.5 eq.) at 0 °C. After being stirred at the same temperature for 15 min, after which total conversion of the starting material into one single product was achieved based on TLC, the mixture was filtered through a small pad of silica gel, washed with ethyl acetate and concentrated in vacuo. The isolated crude trichloroacetimidate (R_f 0.54 (diethyl ether / *n*-pentane (5:95)) was used immediately in the next step without purification.

The crude trichloroacetimidate was transferred into a 10 mL glass pressure microwave tube equipped with a magnetic stirring bar and dissolved in *m*-xylene (2.8 mL). Solid K_2CO_3 (10 mg, 0.070 mmol, 0.1 equiv.) was added, and the mixture was subjected to microwave irradiation at 175 °C for 3 h. After being cooled to room temperature, the insoluble parts were removed by filtration and washed with toluene. The filtrate and toluene washings were concentrated *in vacuo*, and the residue was subjected to column chromatography on silica gel (ethyl acetate / *n*-heptane, from 0:1 to 5:95) to afford the rearranged trichloroacetamide **8** (243 mg, 0.593 mmol, 84%) as a pale yellow oil.

R_f 0.69 (diethyl ether / *n*-pentane (5:95)); **IR** (neat, ν_{max} / cm^{-1}) 3430, 2930, 2860, 1716, 1656, 1498, 1457, 1332, 1220, 1066, 1038, 911, 815, 738, 677; **^{1}H NMR** (400 MHz, $CDCl_3$) δ 6.96 (1H, s, H19), 5.47–4.95 (2H, m, H16), 4.70–4.56 (1H, m, H5), 3.65 (3H, s, H21), 2.49–1.00 (20H, m, H6-15); **^{13}C NMR** (101 MHz, $CDCl_3$) δ 160.8, 153.9, 113.0, 110.9, 93.2, 72.0, 71.3, 69.1, 65.7, 61.6, 57.3, 42.6, 40.1, 38.4, 35.9, 33.5, 32.4, 28.6, 25.5, 25.2, 23.7, 23.4, 23.2, 22.9, 22.8; **HRMS** (ESI+) calc. for $C_{18}H_{27}Cl_3N_2O_2Na$ ($[M+Na]^+$) 431.1030, found 431.1031.

Analysis of the ^{1}H and ^{13}C NMR spectra was complicated by the presence of distinguishable conformers.

2.9. 2,2,2-Trichloro-*N*-(15-methylene-7-azadispiro[5.1.5^8.2^6] pentadecan-14-yl)acetamide-7-*N*-oxyl (9)

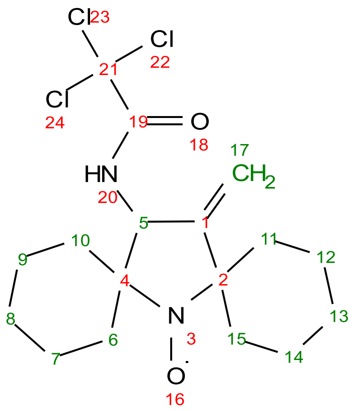

Prepared according to a modified procedure for a related compound.[3] Methoxyamine **8** (250 mg, 0.610 mmol, 1.0 equiv.) was stirred in dichloromethane (6 mL) at room temperature. K_2CO_3 (253 mg, 1.83 mmol, 3.0 equiv.) was added, followed by solid *m*-CPBA (≤77%; 342 mg, 1.52 mmol, 2.5 equiv.) portion-wise over a period of 5 minutes. The reaction was stirred at room temperature for 3 hours. The reaction was then diluted with dichloromethane (5 mL). The organic layer was washed with saturated sodium bicarbonate solution (1 × 10 mL) and water (2 × 10 mL). The organic phase was then dried (Na_2SO_4), filtered and the solvent removed *in vacuo*. The crude mixture was purified by column chromatography (diethyl ether / *n*-pentane (1:9)), providing nitroxide **9** (119 mg, 0.301 mmol, 49%) as an orange solid.

Prior to NMR analysis, radical **9** was reduced *in situ* to the corresponding hydroxylamine by addition of phenylhydrazine directly to the NMR tube.

R_f 0.35 (diethyl ether / *n*-pentane (1:9)); **IR** (neat, ν_{max} / cm^{-1}) 3324, 2928, 2849, 1701, 1528, 1446, 908, 839, 823; **^{1}H NMR** (400 MHz, $CDCl_3$) δ 5.36 (1H, s, H17), 5.18 (1H, s, H17), 4.69 (1H, d, J = 9.4 Hz, H5), 2.05–1.18 (20H, m, H6-15); **^{13}C NMR** (101 MHz, $CDCl_3$) δ 161.1, 151.3, 111.7, 93.1, 69.5, 68.6, 59.7, 40.3, 36.3, 30.5, 29.8, 25.6, 25.4, 23.6, 23.4, 23.1, 23.0; **HRMS** (ESI+) calc. for $C_{17}H_{24}Cl_3N_2O_2Na$ ($[M+Na]^+$) 416.0796, found 416.0799.

2.10. 14-Amino-15-methylene-7-azadispiro[5.1.5^8.2^6]pentadecan-7-*N*-oxyl (10)

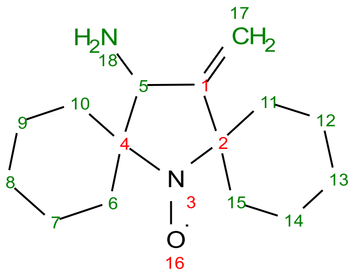

Trichloroacetamide **9** (98 mg, 0.25 mmol, 1.0 equiv.) was dissolved in ethanol (2.5 mL). An aqueous solution (1.2 mL) of KOH (627 mg, 11.2 mmol, 45.0 equiv.) was added slowly with stirring. The reaction flask was equipped with an air condenser and the mixture was stirred for 16 hours at 60 °C. After completion, ethanol was evaporated *in vacuo*. The crude product was diluted with water (5 mL) and acidified with 1 M HCl to pH < 3. The mixture was extracted with diethyl ether (2 × 10 mL). The aqueous fraction was treated with 2 M KOH until reaching pH > 10 and thereafter extracted with ethyl acetate (3 × 10 mL). The organic layers were combined and the solvent was removed *in vacuo*, providing pure amine **10** (59 mg, 0.24 mmol, 95%) as a yellow oil.

Prior to NMR analysis, radical **10** was reduced *in situ* to the corresponding hydroxylamine by addition of phenylhydrazine directly to the NMR tube.

IR (neat, v_{max} / cm^{-1}) 3377, 3373, 2923, 2851, 1663, 1585, 1450, 1417, 1137, 977, 899, 853, 814, 768, 676; **^{1}H NMR** (400 MHz, $CDCl_3$) δ 5.32 (1H, s, H17), 5.24 (1H, s, H17), 3.73 (1H, s, H5), 2.11–1.27 (22H, m, H6-15, H18); **^{13}C NMR** (101 MHz, $CDCl_3$) δ 113.0, 59.8, 40.1, 30.4, 29.8, 29.7, 25.7, 25.4, 23.4, 23.3, 23.2.; **HRMS** (ESI+) calc. for $C_{15}H_{25}N_2ONa$ ($[M+Na]^+$) 272.1859, found 272.1859.

Significant line-broadening is apparent in the ^{13}C NMR spectrum due to low concentration of the sample and complex mixture after reduction with phenylhydrazine. Thus, not all peaks were possible to assign.

2.11. 2-Chloro-*N*-(15-methylene-7-azadispiro[5.1.5⁸.2⁶]pentadecan-14-yl)acetamide-7-*N*-oxyl (11)

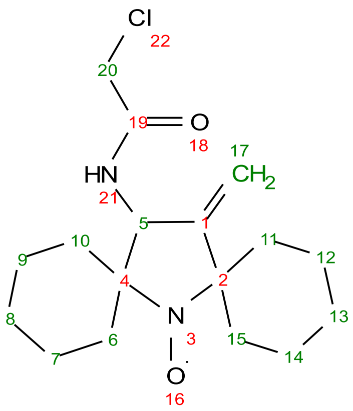

Prepared according to a modified procedure for the preparation of a related compound.[5] To a solution of amine **10** (59 mg, 0.24 mmol, 1.0 equiv.) in dry THF (0.8 mL) was added dry triethylamine (66 µL, 0.47 mmol, 2.0 equiv.) under argon and the mixture was cooled down to 0 °C. Chloroacetyl chloride (31 µL, 0.39 mmol, 1.6 equiv.) was added under argon at 0 °C and the mixture was stirred at room temperature for 1 h. After that, the mixture was diluted with THF (5 mL) and then filtered through cotton to remove the white precipitate. The filtrate was concentrated *in vacuo*. The residue was redissolved in dichloromethane (5 mL) and washed with brine (3 × 10 mL). The organic layer was dried with Na_2SO_4 and concentrated. Purification of the crude material on silica gel flash column chromatography (diethyl ether / dichloromethane (1:9)) gave chloroacetamide **11** (48 mg, 0.15 mmol, 62%) as a yellow oil.

Prior to NMR analysis, radical **11** was reduced *in situ* to the corresponding hydroxylamine by addition of phenylhydrazine directly to the NMR tube.

R_f 0.68 (diethyl ether / dichloromethane (1:9)); **IR** (neat, ν_{max} / cm^{-1}) 3304, 2930, 2858, 1656, 1528, 1451, 1415, 1356, 1265, 1150, 905, 786; **^{1}H NMR** (400 MHz, $CDCl_3$) δ 5.28 (1H, s, H17), 5.17 (1H, s, H17), 4.84 (1H, d, *J* = 9.8 Hz, H5), 4.16–4.06 (2H, m, H20), 2.12–1.03 (20H, m, H6-15); **^{13}C NMR** (101 MHz, $CDCl_3$) δ 42.9, 25.5, 25.3, 23.4, 23.0; **HRMS** (ESI+) calc. for $C_{17}H_{26}ClN_2O_2Na$ ($[M+Na]^+$) 348.1575, found 348.1575.

Significant line-broadening is apparent in the ^{13}C NMR spectrum due to low concentration of the sample and complex mixture after reduction with phenylhydrazine. Thus, not all the peaks were possible to assign.

2.12. 2-Iodo-*N*-(15-methylene-7-azadispiro[5.1.5^{8}.2^{6}]pentadecan-14-yl)acetamide-7-*N*-oxyl (12)

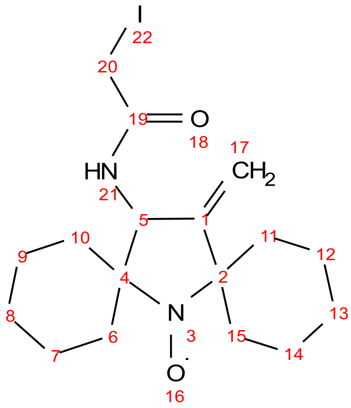

Caution: Sodium iodide and the product are light sensitive! Avoidance of light is advised at any stage of the reaction, purification and storage.

Prepared according to a modified procedure for the preparation of a related compound.[5] The chloroacetamide **11** (14 mg, 43 µmol, 1.0 equiv.) and dry sodium iodide (19 mg, 0.13 mmol, 2.9 equiv.) were placed in a Schlenk flask covered by aluminium foil. The flask was evacuated and back-filled with argon, and dry acetone (0.34 mL) was added. The mixture was stirred at room temperature for 1 hour. After that, the mixture was concentrated *in vacuo* and the residue was dissolved in dichloromethane (3 mL), washed with brine (10 mL), dried with Na_2SO_4 and evaporated. Purification of the crude material by silica gel flash column chromatography (diethyl ether / dichloromethane from 0:1 to 5:95) gave the spin label **12** (16 mg, 38 µmol, 89%) as a yellow solid.

R_f 0.40 (diethyl ether / dichloromethane (5:95)); **IR** (thin film, ν_{max} / cm^{-1}) 3292, 2927, 2854, 1650, 1541, 1450, 1421, 1342, 1168, 910; **HRMS** (ESI+) calc. for $C_{17}H_{26}IN_2O_2Na$ ($[M+Na]^+$) 440.0931, found 440.0931.

Due to the high sensitivity of the product and possible unwanted reactivity towards phenylhydrazine, this compound was not analysed by NMR spectroscopy.

3. Crystallography data

Crystals of nitroxide **9** were grown by slow evaporation of a solution of the compound in diethyl ether. X-ray diffraction data were collected at 100 K using a Rigaku MM-007HF High Brilliance RA generator/confocal optics with XtaLAB P200 diffractometer [Cu Kα radiation (λ = 1.54187 Å)]. Data were collected (using a calculated strategy) and processed (including correction for Lorentz, polarization and absorption) using CrysAlisPro.[6] Structure was solved by dual-space methods (SHELXT)[7] and refined by full-matrix least-squares against F^2 (SHELXL-2019/3).[8] Non-hydrogen atoms were refined anisotropically, and hydrogen atoms were refined using a riding model except for the hydrogen atom on N12 which was located from the difference Fourier map and refined isotropically subject to a distance restraint. All calculations were performed using the Olex2 interface.[9] Selected crystallographic data are presented in Table S1. CCDC 2406207 contains the supplementary crystallographic data for this paper. These data can be obtained free of charge from The Cambridge Crystallographic Data Centre via www.ccdc.cam.ac.uk/structures.

Table S1. Selected crystallographic data.

	**9**
formula	$C_{17}H_{24}Cl_3N_2O_2$
fw	394.73
temperature [K]	100
crystal description	Yellow needle
crystal size [mm^3]	0.19 × 0.02 × 0.01
space group	$P2_1/c$
a [Å]	12.68805(19)
b [Å]	14.41773(19)
c [Å]	10.39362(16)
β [°]	102.5135(15)
vol [Å]^3	1856.17(5)
Z	4
ρ (calc) [g/cm^3]	1.413
μ [mm^{-1}]	4.572
F(000)	828
reflections collected	19950
independent reflections (R_{int})	3799 (0.0626)
parameters, restraints	221, 1
GoF on F^2	1.079
R_1 [$I > 2\sigma(I)$]s	0.0376
wR_2 (all data)	0.1064
largest diff. peak/hole [e/Å^3]	0.60/-0.36

4. Preparation of spin-labelled protein

4.1. Preparation of CaM/M13 34C 146C

The construct expression and protein purification were done as presented elsewhere.[10] The double cysteine mutant of CaM/M13 was prepared as detailed therein.[10]

4.2. Visualisation of CaM/M13 34C 146C

The 34C 146C mutant of CaM/M13 was visualised in PyMOL (Figure 1 in the main manuscript), based on PDB ID 2BBM.[11, 12] The Mutagenesis Wizard was used to perform the T34C T146C mutations *in silico*. For each residue the lowest energy conformer of the cysteine side chains were chosen, except when two conformers had approximately equal strain energies and the slightly higher energy conformer gave much better visibility of the mutation.

4.3. Spin labelling

To a 50 µM solution of CaM/M13 in 500 µL of 40 mM HEPES and 150 mM NaCl buffer, pH 7.5, was added 10 equivalents of spin label **12** (from a 20 mM stock solution in DMSO) to a final concentration of 500 µM **12**. The mixture was incubated in darkness, with gentle shaking, at 4 °C, overnight (~ 16 h). TOF mass spectrometry analysis (Waters Xevo G2TOF with Waters Acquity LC) with ESI+ ionisation was performed to assess the efficiency of the labelling process, revealing a mixture of singly- and doubly-labelled protein in an approximate 3:10 ratio (Figure S1). The expected mass of the doubly-labelled CaM/M13 was 22513.3 Da and the measured mass was 22513.5 Da.

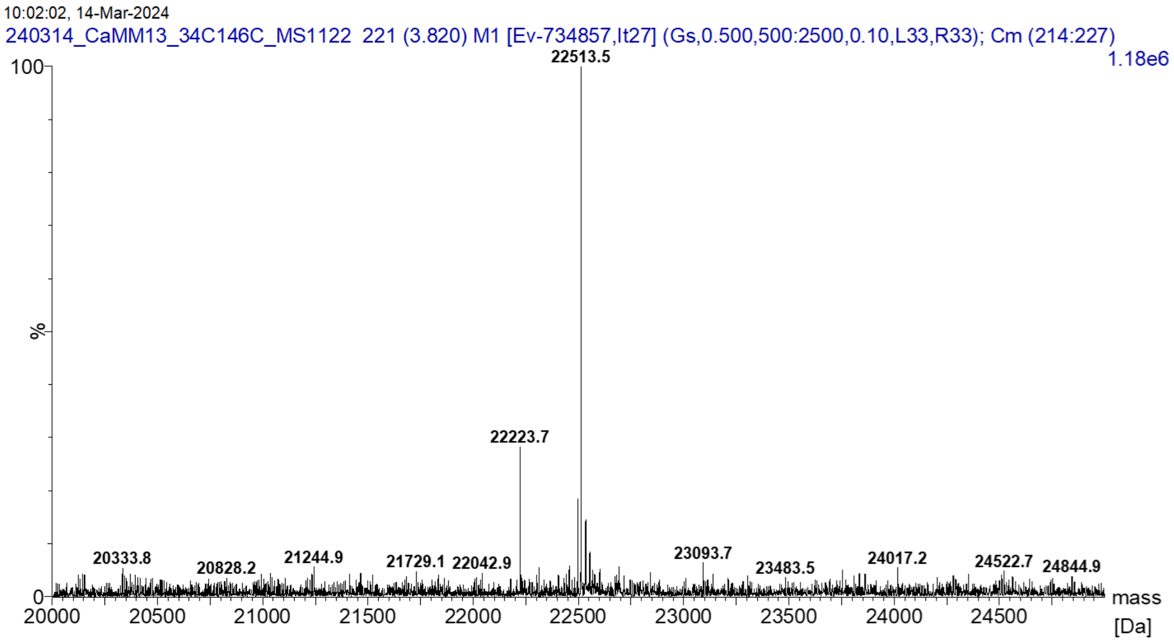

Figure S1. MS spectrum of labelled CaM/M13. 22223.7 Da indicates singly labelled protein and 22513.5 Da doubly-labelled protein.

Purification by treatment with biotin-maleimide and affinity chromatography with a streptavidin column was performed as previously described,[10] using 40 mM HEPES and 150 mM NaCl buffer, pH 7.5, yielding pure doubly-labelled CaM/M13 protein (Figure S2). The sample was transferred to D_2O buffer (40 mM HEPES and 150 mM NaCl, pH 7.5) using a Bio-Spin P6 Gel column. The protein concentration was determined through UV-Vis spectroscopy, using the Beer-Lambert law. The extinction coefficient of CaM/M13 34C 146C construct used was 8605 $M^{-1}cm^{-1}$ and the light path of the cuvette was 0.1 cm. The solution corresponding to Sample 1 (see section 5.4) was measured immediately. The solution for Sample 2 was stored at 253 K.

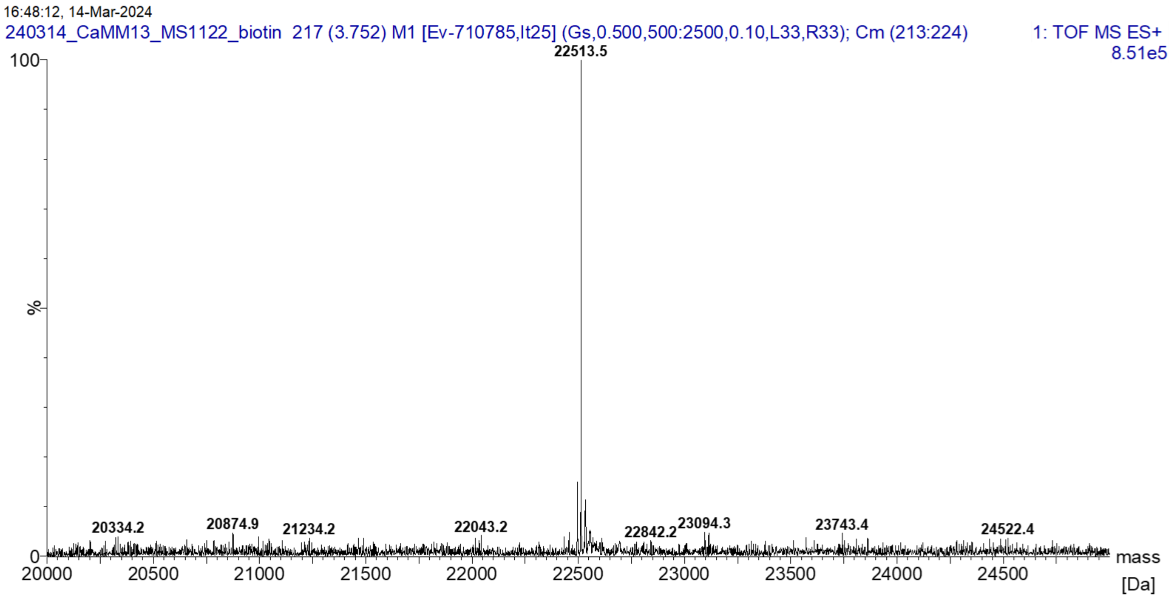

Figure S2. MS spectrum of doubly-labelled CaM/M13 after purification.

5. EPR spectroscopy

5.1. CW spectra of nitroxides and labelled protein

Room temperature CW EPR measurements were acquired using an X-band Affirmo microESR Benchtop EPR Spectrometer.

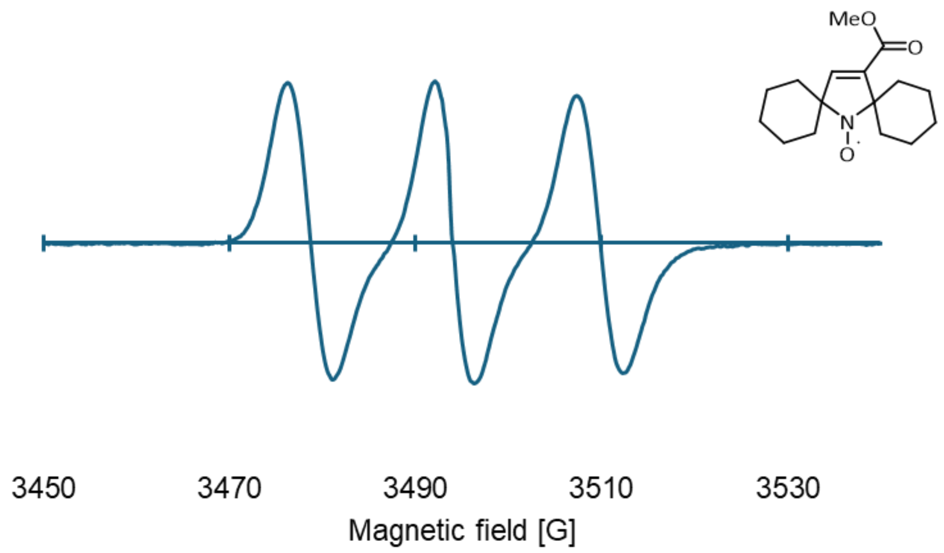

Figure S3. Room temperature X-band (9.81 GHz) CW EPR spectrum of nitroxide **5** (2 mM in PBS buffer/DMSO 1:1 v/v).

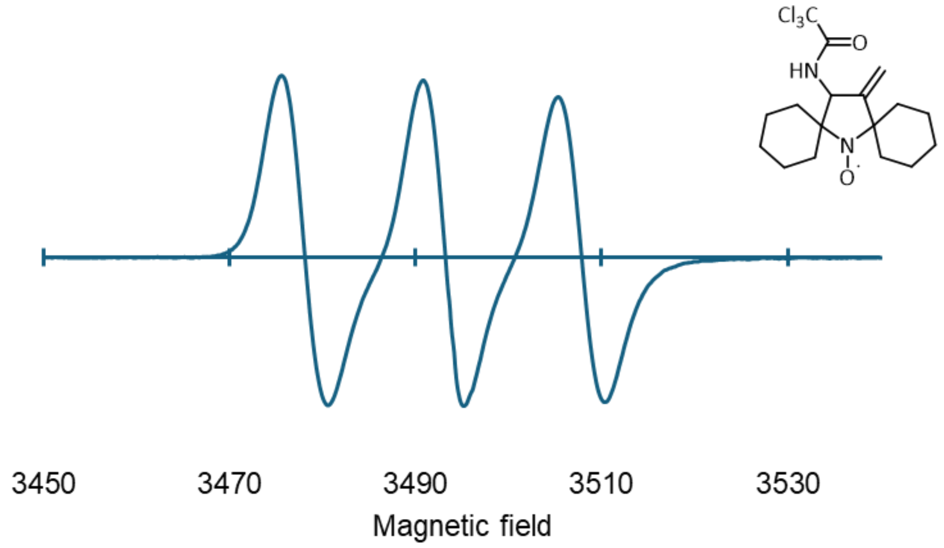

Figure S4. Room temperature X-band (9.81 GHz) CW EPR spectrum of nitroxide **9** (2 mM in DMSO).

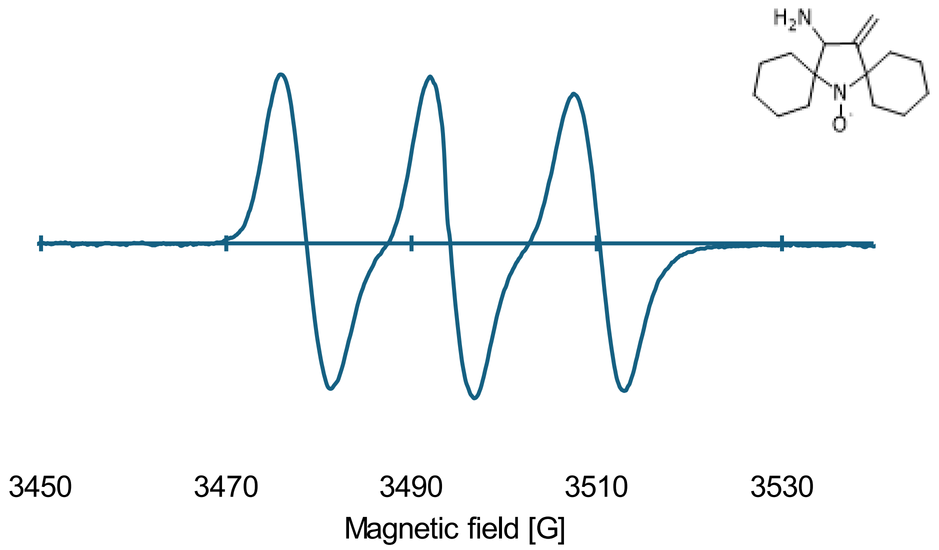

Figure S5. Room temperature X-band (9.81 GHz) CW EPR spectrum of nitroxide **10** (2 mM in PBS buffer/DMSO 1:1 v/v).

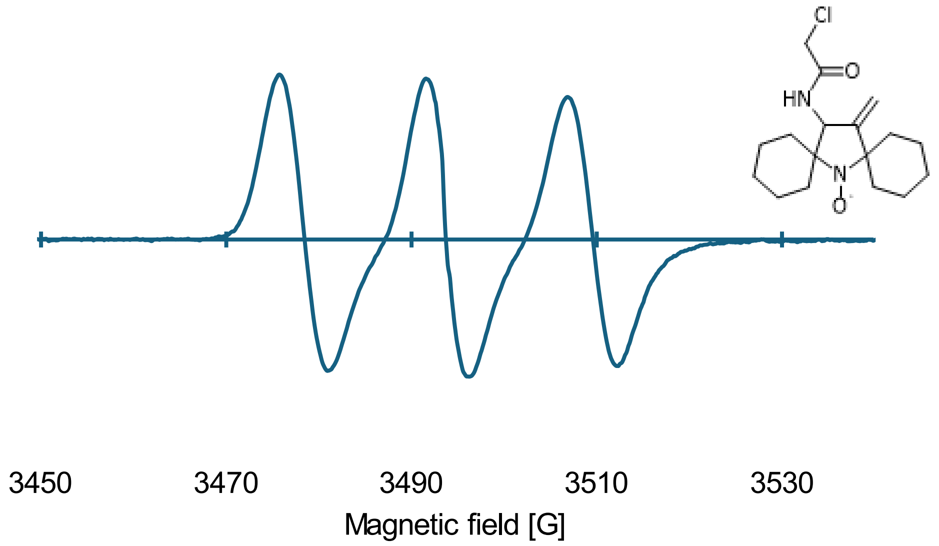

Figure S6. Room temperature X-band (9.81 GHz) CW EPR spectrum of nitroxide **11** (2 mM in PBS buffer/DMSO 1:1 v/v).

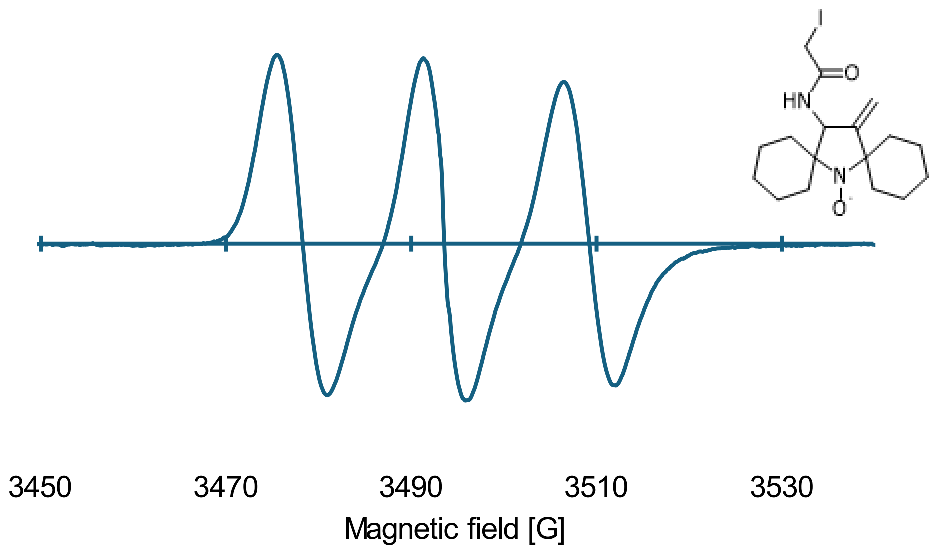

Figure S7. Room temperature X-band (9.81 GHz) CW EPR spectrum of nitroxide **12** (2 mM in PBS buffer/DMSO 1:1 v/v).

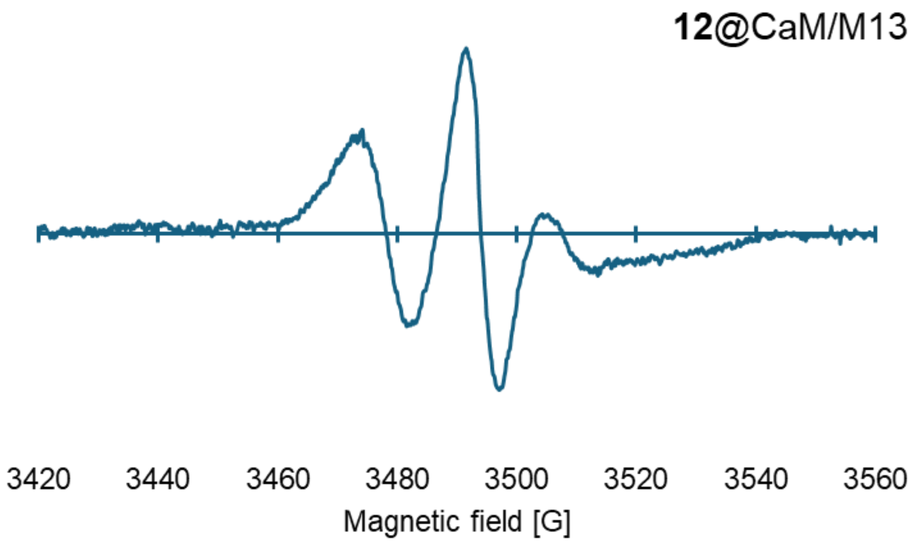

Figure S8. Room temperature X-band (9.80 GHz) CW EPR spectrum of CaM/M13 34C 146C labelled with nitroxide **12** (250 µM in HEPES buffer, pH 7.5).

5.2. Kinetic studies

A solution of 100 mM sodium ascorbate in 40 mM HEPES and 150 mM NaCl buffer, pH 7.5 was prepared. 30 µL of a 250 µM labelled protein solution in the previously described HEPES buffer was transferred by micropipette to an Eppendorf tube. 2.4 µL of the 100 mM ascorbate solution was added corresponding to a 32-equiv. excess over protein (16 times over [**12**]), and the timer was started as the tube was gently mixed by slow aspiration and dispensing of the mixture from the micropipette. The resultant mixture was drawn into a capillary tube, stoppered with clay, and placed into the EPR sample cavity in the benchtop spectrometer. The peak height of the low-field line of the nitroxide triplet was measured as a function of time. EPR parameters: Microwave power 10 mW, modulation coil amplitude 100%, field range 3400–3588 G, number of points 1600, sweep time 9.3 s. Decay over time is presented in Figure 2 in the manuscript.

5.3. Relaxation measurements

1 µL of a 100 mM **12** solution in DMSO was placed in an Eppendorf tube. 999 µL of PBS buffer was added, resulting in a 100 µM solution of nitroxide in PBS buffer/DMSO 999:1 v/v. 30 µL of this solution was transferred to an Eppendorf tube and diluted with 30 µL of glycerol, resulting in a 50 µM solution of **12** in glycerol/PBS buffer/DMSO 1000:999:1 v/v. For CaM/M13 34C 146C labelled with **12** the sample was 80 µM protein in 40 mM HEPES, 150 mM NaCl, 10 mM $CaCl_2$ pH 7.5 in D_2O, with 50% glycerol-d_8 (v/v) added just prior to measurement. The sample solution was placed in a quartz EPR tube (approximately 2.9 mm outer diameter) and cooled in liquid nitrogen prior to analysis by EPR spectroscopy. A Bruker Elexsys E580 with high powered (150 W) Q-band (34 GHz) and an ER 5106QT-2w cylindrical resonator was used. T_m data was collected by monitoring the echo decay at the maximum intensity of the nitroxide signal using a $\frac{\pi}{2} - \tau - \pi$ pulse sequence with 16/32 ns pulses and an initial τ of 380 ns. 500 points were taken with $\Delta\tau$ set 20 ns (H_2O 50 K, 60 K, 80 K, 100 K, 180 K) or 40 ns (D_2O and H_2O 120–160 K). The shot repetition time was 50 ms (50 K), 20 ms (60 K, 80 K), 10 ms (100 K), 5 ms (120, 140, 160 K, D_2O 180 K), 2 ms (H_2O 180 K), the whole echo was integrated over, and a 2-step phase cycle was used. Data were taken with 5 shots per point. The resonator was overcoupled as for DEER measurements and was adjusted at 120 K, 140 K (protein sample) or 160 K (spin label solution), and 180 K. The temperature was incremented from 50 K upwards with at least a few minutes at each new temperature to allow stabilisation of the temperature. The measurements were only taken once (no repeats) and not to equal signal-to-noise ratio. There is also a deadtime associated with the initial value of τ, so the phase memory times presented in this paper are only indicative, not fully accurate. Indicative curves

are shown in Figure 3 of the main paper, where the time axis is the total incremented time from the first pulse.

Relaxation times were extracted using the curve fitting tool in Matlab. For simplicity of analysis, phase memory time T_m data were fitted to the function $y = a \cdot e^{-\left(\frac{t}{T_m}\right)^c + d}$, although this was not always necessary (indicated for cases where the stretch parameter is almost unity). However, the extracted values give a good indication of the overall trend in relaxation times, dependent on molecule and temperature. The log of the reciprocal of the T_m values and the stretch parameters are given in Figure S9A and B, respectively.

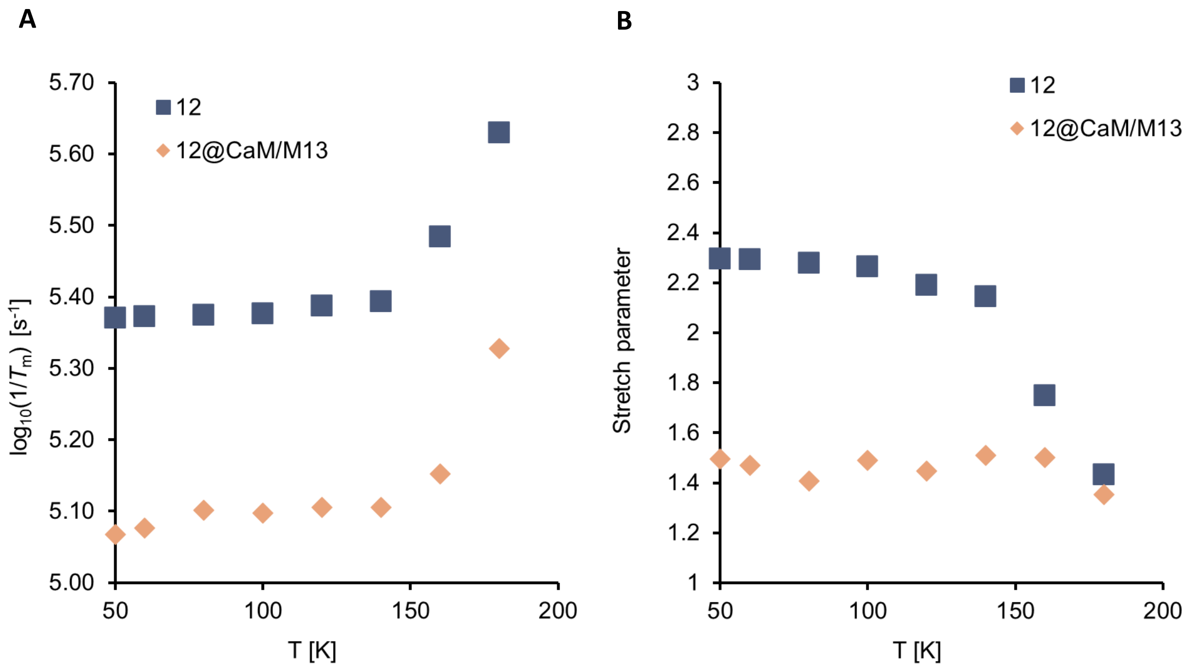

Figure S9. A) Plots of the phase memory time T_m, measured through spin echo decay, for the nitroxide **12** and **12**-labelled **CaM/M13**, versus temperature. **B)** Stretch parameter for fitting the spin echo decay curve.

5.4. DEER experiment

The DEER samples were prepared over two batches: Sample 1 and Sample 2. Sample 1 was described previously in section 5.3 and the DEER measurements were taken in a 2.9 mm OD quartz tube as the sample temperature was 50 K or 120 K. Sample 2 was prepared at the same concentration and the same solution as Sample 1, then 40 µL was transferred to a fluorinated ethylene propylene (FEP) tube which was then inserted into the 2.9 mm OD quartz tube. The FEP tube allowed for a DEER measurement at 180 K where we previously had experienced a broken quartz tube when measuring Sample 1 at this temperature. However, using the FEP tube resulted in a smaller sample volume and led us to measure a shorter DEER time trace with less good signal-to-noise ratio (SNR) in the available time.

The DEER experiments were carried out with the same spectrometer as in Section 5.3. The four-pulse DEER sequence was: $(\frac{\pi}{2})_{\mathrm{obs}} - \tau_1 - \pi_{obs} - (\tau_1 + t) - \pi_{\mathrm{pump}} - (\tau_2 - t) - \pi_{\mathrm{obs}} - \tau_2 - \mathrm{echo}$.[13-16] The pump frequency was set to the maximum of the nitroxide spectral intensity and corresponding to the minimum in the cavity coupling response (approximately 34 GHz). The observer (“obs”) echo sequence was set at 80 MHz lower frequency. The other parameters were: time step t = 8 ns, starting at – 320 ns and going until 80 ns before the final pulse; τ_1 = 400 ns with a 5 step average where 24 ns was added to the previous value each time; 30 shots per point averaged by the spectrometer; pump pulse length 12 ns (Sample 1, 50 K), 20 ns (Sample 1, 120 K), 14 ns (Sample 2, 50 K), 16 ns (Sample 2, 120 K and 180 K); Observer pulse lengths 10/20 ns (Sample 1, 50 K; Sample 2, 120 K and 180 K), 7/14 ns (Sample 1, 120 K), 13/26 ns (Sample 2, 50 K); τ_2 = 8 µs (Sample 1), τ_2 = 5 µs (Sample 2); shot repetition time set to recover about 80% of the echo 5 ms (Sample 1, 50 K), 1 ms (Sample 1 and 2, 120 K), 4 ms (Sample 2, 50 K), 0.5 ms (Sample 2, 180 K); averages = 5 (Sample 1, 50 K), 33 (Sample 1, 120 K), 1 (Sample 2, 50 K), 7 (Sample 2, 120 K), 52 (Sample 2, 180 K). A 16-step phase cycle was used which was necessary due to using the AWG channel. For Sample 1 the DEER time trace data are shown in Figure S10A. For Sample 2 the DEER time traces are shown in Figure S11A. Note that for the 180 K data a severe (linear) drift in the phase was noted over the night, these data were analysed regardless of this phase drift.

The data were analysed to provide distance distributions using DEERNet v2.8 with first removing 800 ns of the final data points of the raw data (already removed in the displayed time traces).[17, 18] These are shown for Sample 1 in Figure S10B and for Sample 2 in Figure S11B.

The data were also analysed with DeerAnalysis2022 (MatLab 2023a) by cutting the last 800 ns and allowing the software to determine the zero time, background correction start time and phase correction.[19] Tikhonov regularization was then used with the regularization parameter determined through the automatic L-curve corner determination. These are reported alongside

the background corrected DEER time traces, fits and resulting distance distributions in Figure S12.

The distance distributions for the Sample 1 and Sample 2 at 50 K do not perfectly align (compare the blue lines in Figure S10B and Figure S11B or Figure S12B) which we believe to be a consequence of a lower SNR and shorter time trace for Sample 2, but also possibly due to the storage of the protein in between the measurements. We stored several hundred µL protein solution in the freezer without glycerol, and this may have led to a slight change in conformation – an effect we have occasionally seen with this protein. There is slight variation, though mostly within the DEERNet 95% confidence level for the data taken at 50 and 120 K for each sample. At 180 K the deviation is slightly greater, but given the lower SNR for this measurement we do not interpret this as a change of conformation in the softening glass.

Figure S13 shows the distance distribution from Sample 1 at 120 K, as shown in the main paper (Figure 4B). The result is compared to the chiLife model for the MTSL spin label at positions 34 and 146 of the 2BBM PDB structure (which we hypothesise to closely resemble our CaM/M13 structure).[12, 20] The model predicts the longer distance of the distribution quite well (4.24 nm for the most probable distance from the model compared to 4.17 nm from the DEER experiment), assuming that using MTSL as a proxy for **12** is reasonable. The MTSL experimental result on this protein variant has been published elsewhere.[10] The MTSL on CaM/M13 34C 146C with Ca^{2+} has a very narrow distribution which does not overlap with the chiLife model prediction, but tentatively we assign it to the highest amplitude peak in the distance distribution of CaM/M13 labelled with **12** (3.62 nm for the most probable distance for the MTS-labelled protein compared to 3.59 nm for the protein labelled with **12**). These results taken together therefore suggest that our proposed model protein system has a complicated energy landscape for the spin labels and that this may be the underlying factor for the broader distribution of spin label **12** on CaM/M13 than we had anticipated.

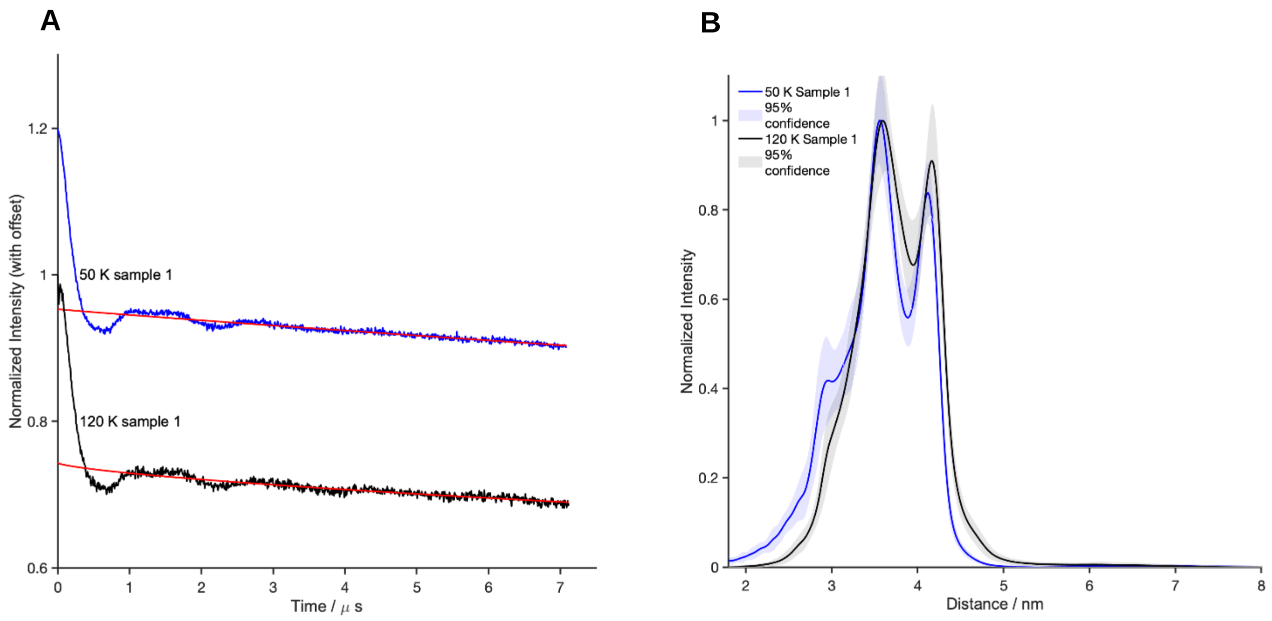

Figure S10. DEERNet results for Sample 1. **A)** DEER time trace data at 50 and 120 K. The red line indicates the background function found by DEERNet. **B)** Distance distributions.

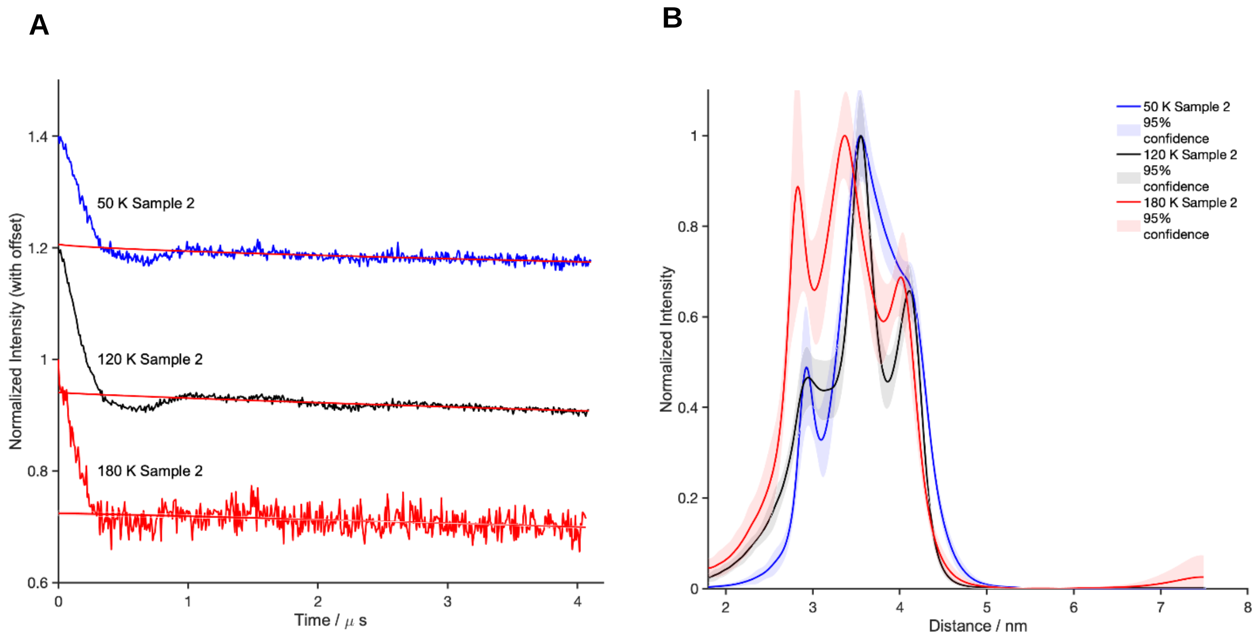

Figure S11. DEERNet results for Sample 2. **A)** DEER time trace data at 50, 120 and 180 K. The red line indicates the background function determined by DEERNet. **B)** Distance distributions.

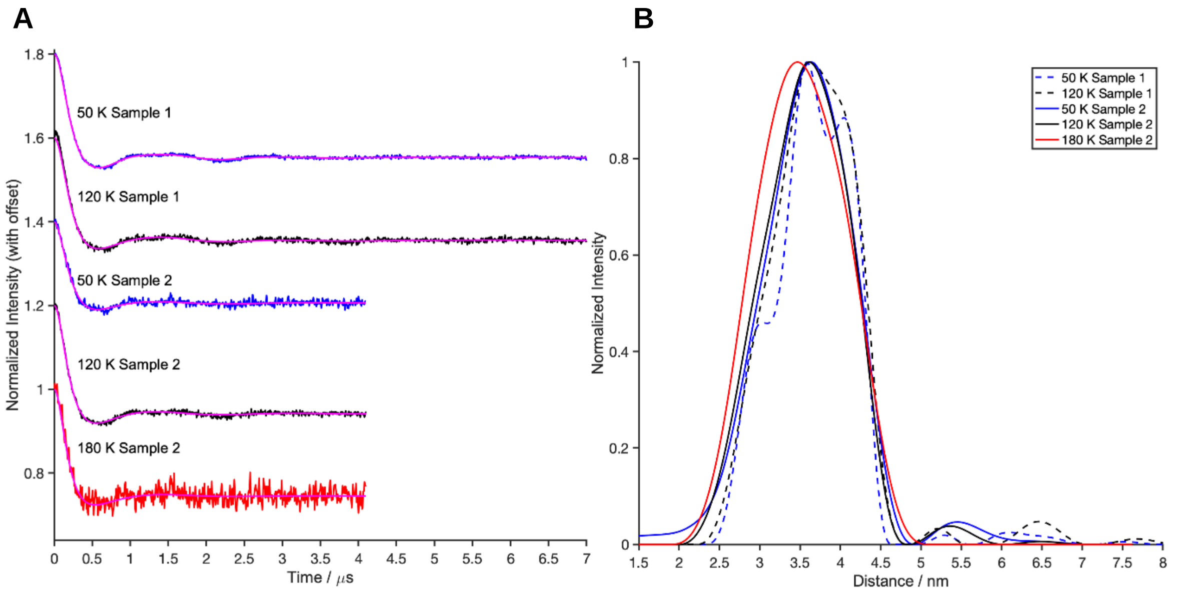

Figure S12. DeerAnalysis results. **A)** DEER time trace data (after background correction). The magenta line is the fit from DeerAnalysis. **B)** Distance distributions. The Tikhonov regularisation parameters were: 1585 (Sample 1, 50 K); 3981 (Sample 1, 120 K); 3162 (Sample 2, 50 K); 2512 (Sample 2, 120 K); 5012 (Sample 2, 180 K).

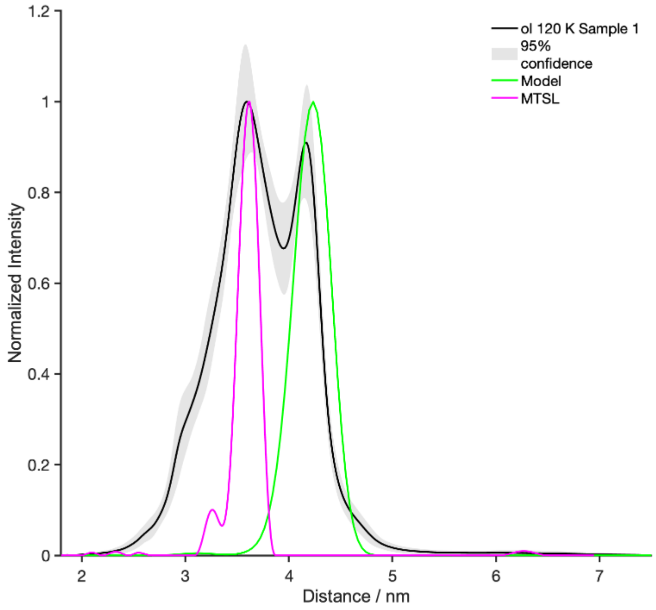

Figure S13. Overlay of DEERNet distance distribution for Sample 1 measured at 120 K (black) with prediction acquired with the chiLife software (green) and compared to the same protein labelled with MTSL (magenta; data from Ref. 10).

NMR spectra

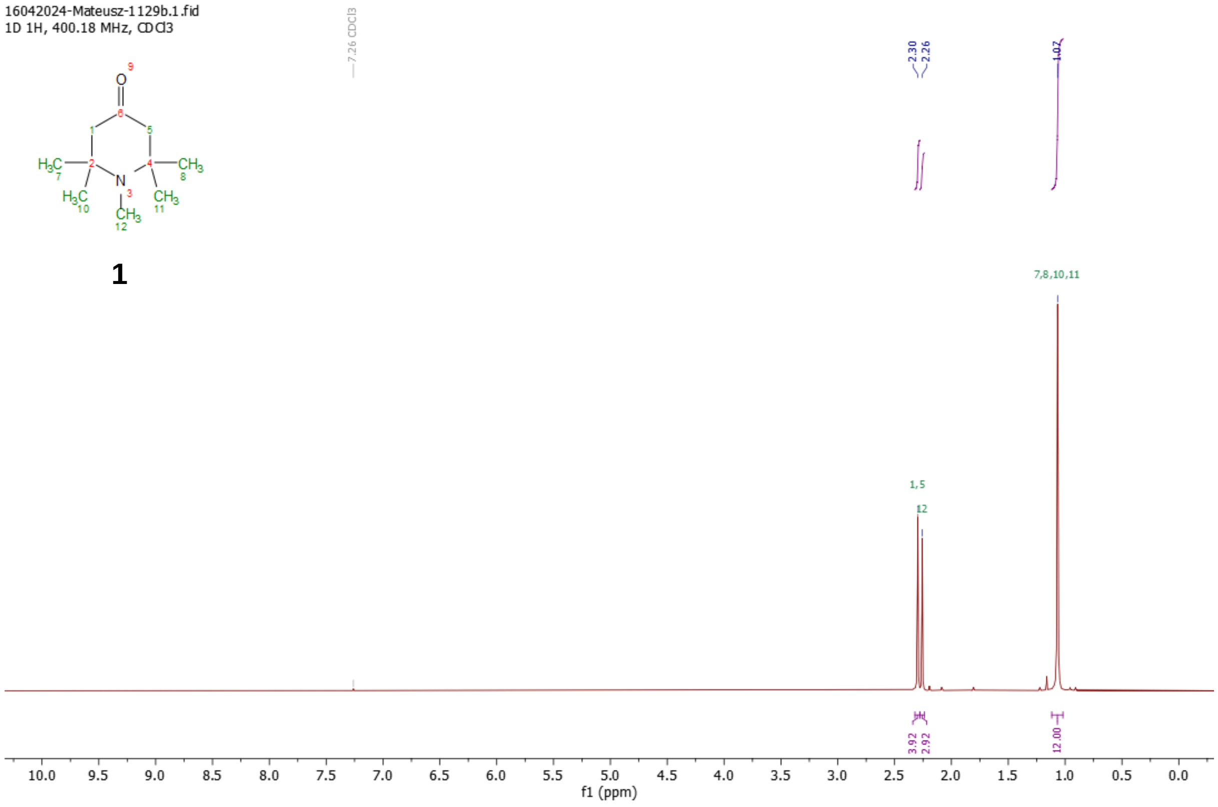

^{1}**H NMR** (400 MHz, $CDCl_3$) δ 2.30 (4H, s, H1, H5), 2.26 (3H, s, H12), 1.07 (12H, s, H7, H8, H10, H11).

Figure S14. ^{1}H NMR spectrum of **1**.

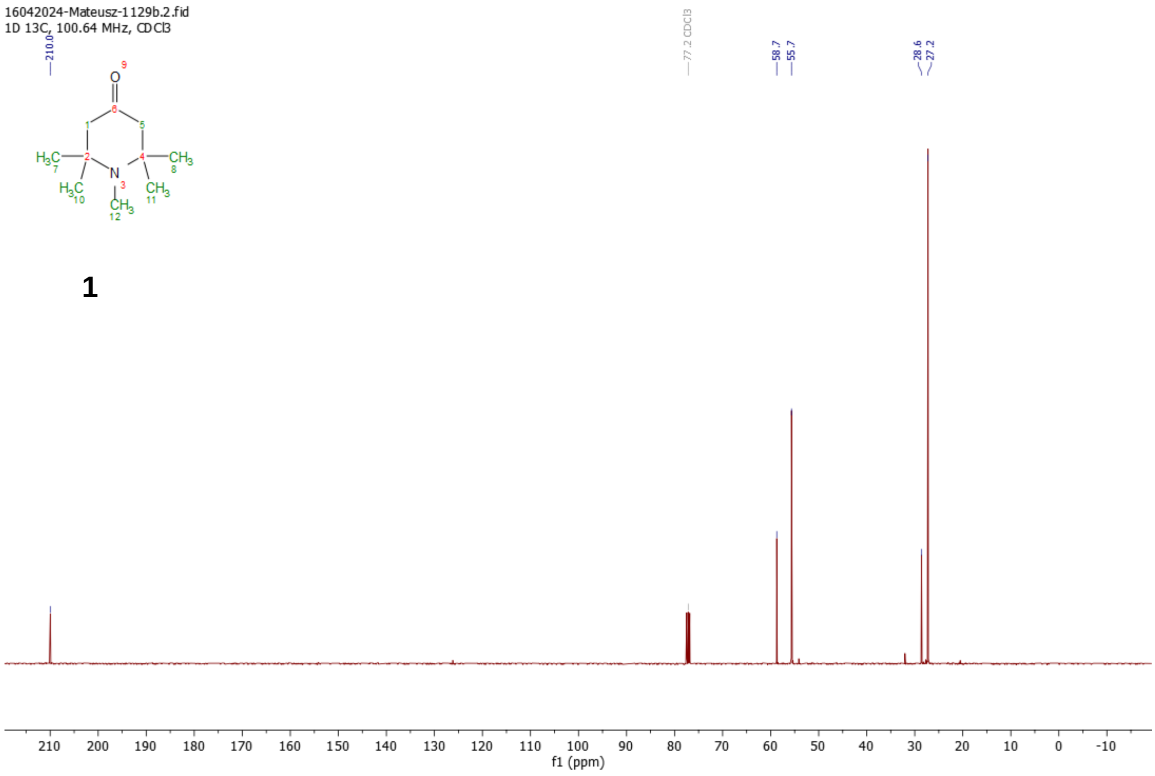

^{13}C NMR (101 MHz, $CDCl_3$) δ 210.0, 58.7, 55.7, 28.6, 27.2.

Figure S15. ^{13}C NMR spectrum of **1**.

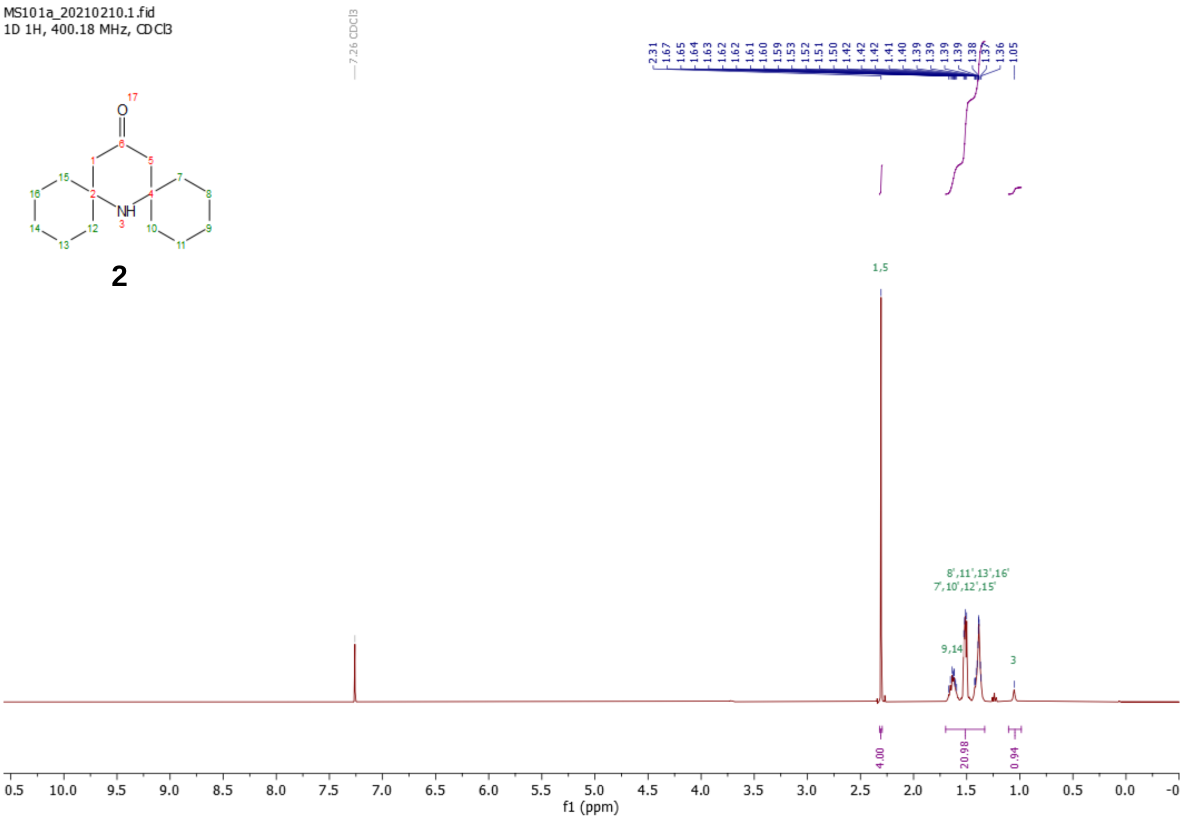

[1]**H NMR** (400 MHz, $CDCl_3$) δ 2.31 (4H, s, H1, H5), 1.70–1.33 (20H, m, H7, H8, H9, H10, H11, H12, H13, H14, H15, H16), 1.05 (1H, s, H3).

Figure S16. ^{1}H NMR spectrum of **2**.

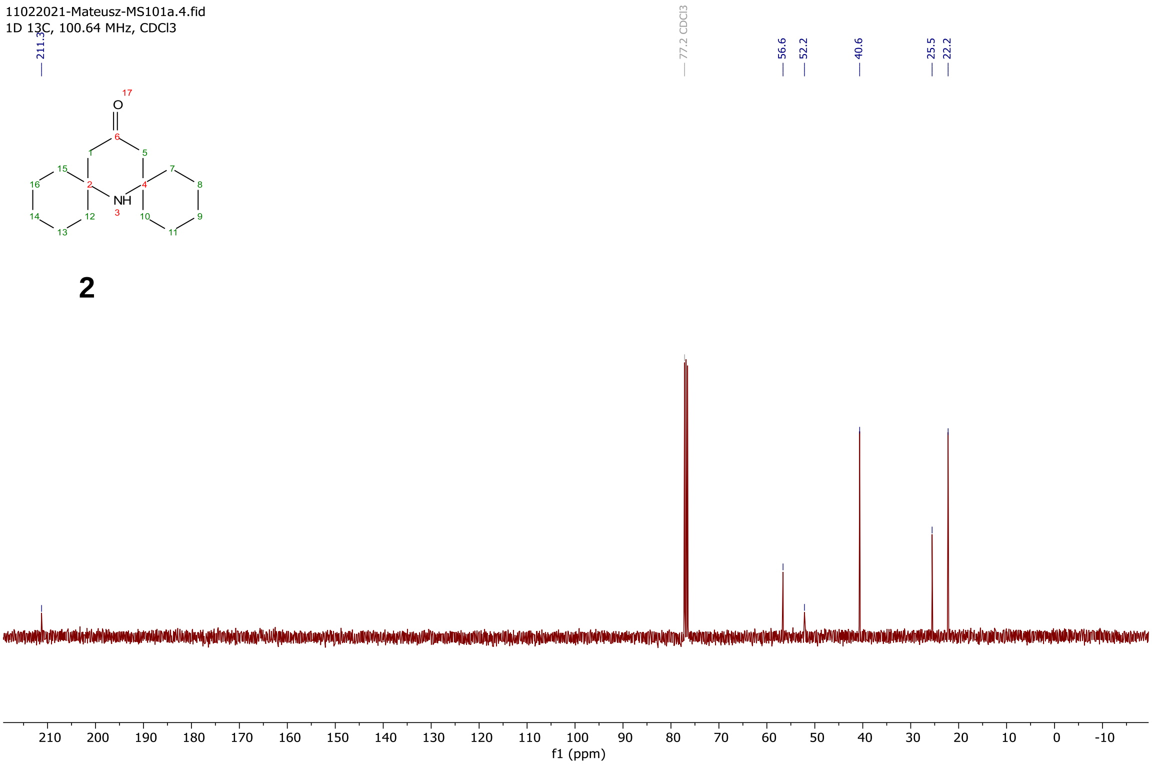

[13]**C NMR** (101 MHz, $CDCl_3$) δ 211.3, 56.6, 52.2, 40.6, 25.5, 22.2.

Figure S17. ^{13}C NMR spectrum of **2**.

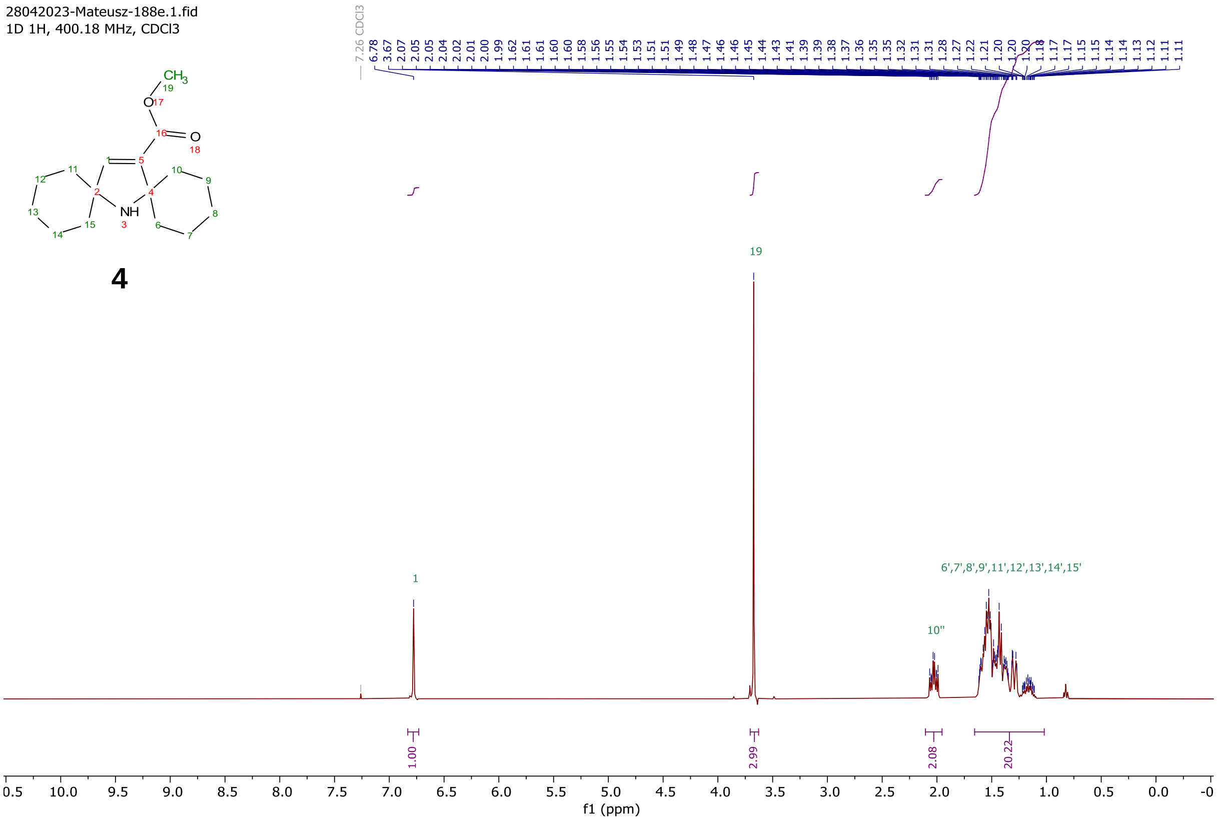

^{1}**H NMR** (400 MHz, $CDCl_3$) δ 6.78 (1H, s, H1), 3.67 (3H, s, H19), 2.03 (2H, td, *J* = 12.7, 5.7 Hz, 10''), 1.66–1.02 (20H, m, 6', 7', 8', 9', 11', 12', 13', 14', 15').

Figure S18. ^{1}H NMR spectrum of **4**.

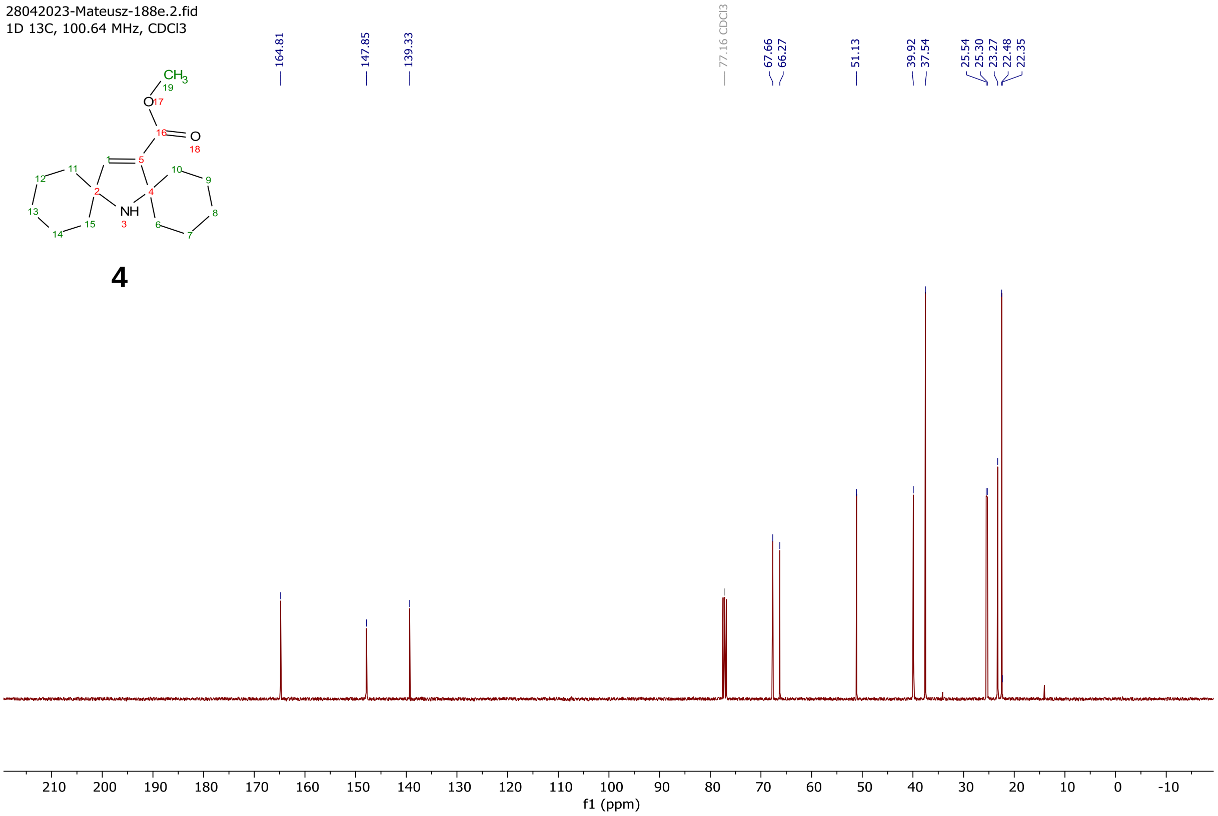

^{13}**C NMR** (101 MHz, $CDCl_3$) δ 164.8, 147.8, 139.3, 67.7, 66.3, 51.1, 39.9, 37.5, 25.5, 25.3, 23.3, 22.5, 22.3.

Figure S19. ^{13}C NMR spectrum of **4**.

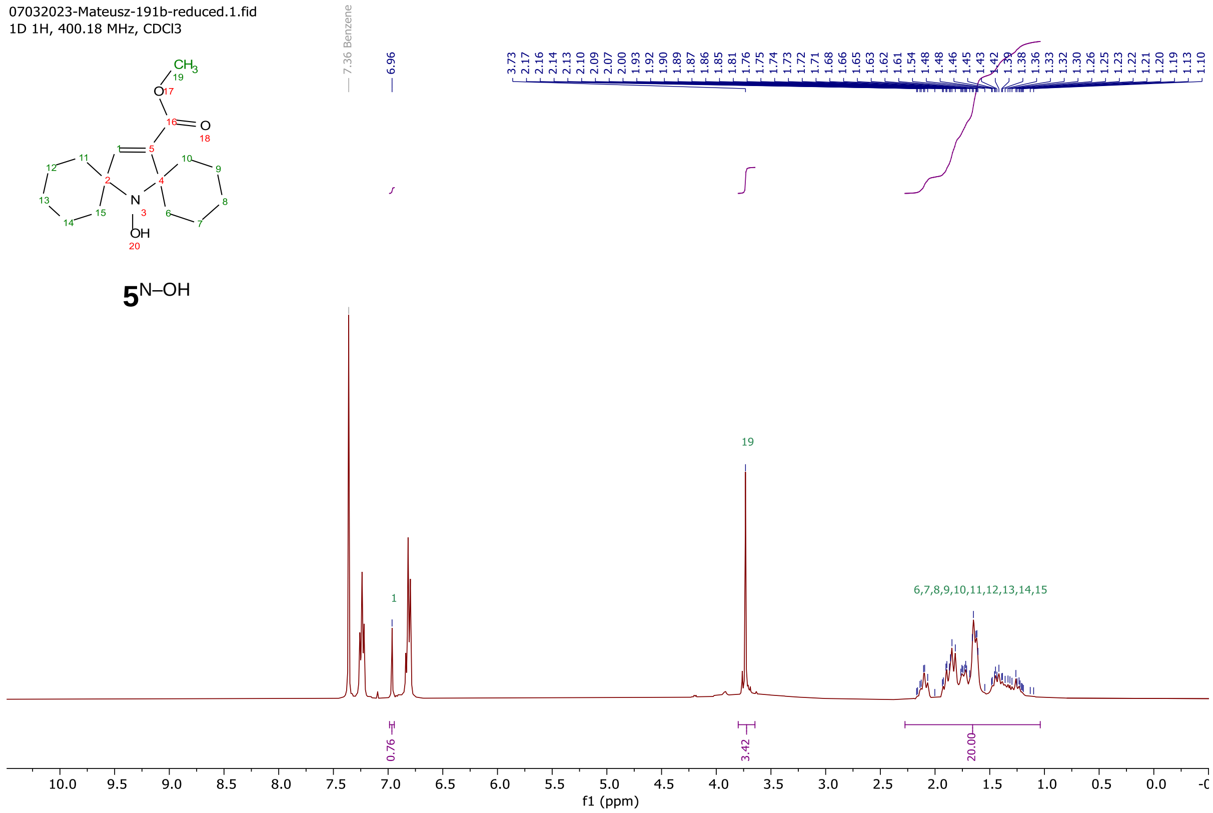

¹H NMR (400 MHz, $CDCl_3$) δ 6.96 (1H, s, H1), 3.73 (3H, s, H19), 2.27–1.04 (20H, m, H6, H7, H8, H9, H10, H11, H12, H13, H14, H15).

Figure S20. ^{1}H NMR spectrum of **5** after addition of phenylhydrazine.

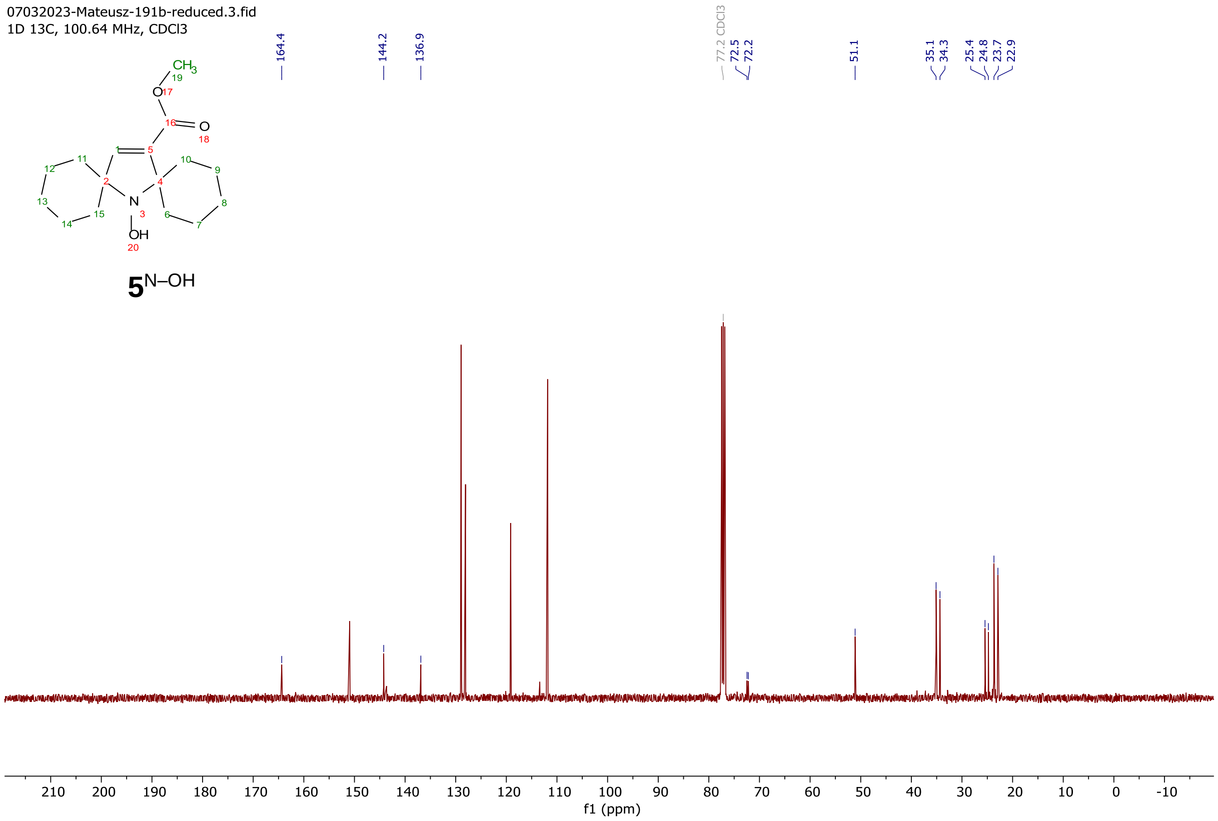

¹³C NMR (101 MHz, $CDCl_3$) δ 164.4, 144.2, 136.9, 72.5, 72.2, 51.1, 35.1, 34.3, 25.4, 24.8, 23.7, 22.9.

Figure S21. ^{13}C NMR spectrum of **5** after addition of phenylhydrazine.

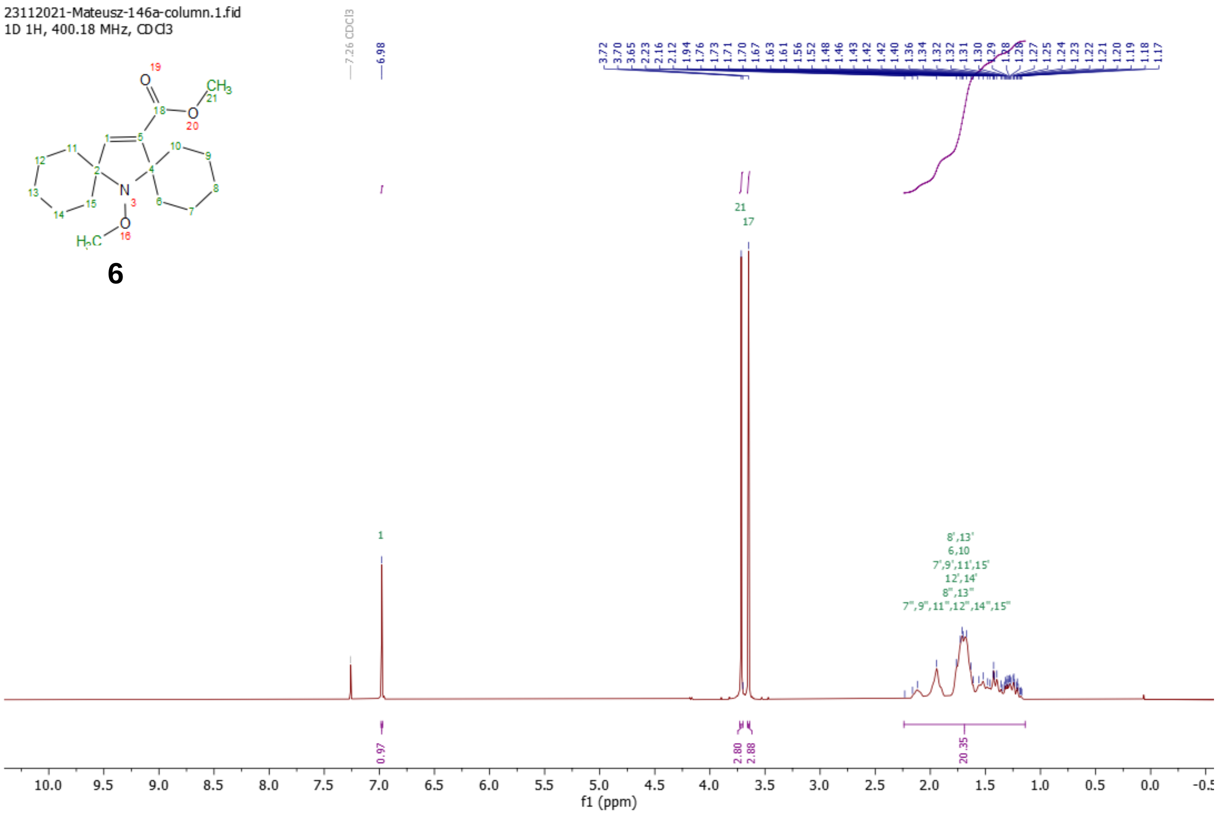

^{1}**H NMR** (400 MHz, $CDCl_3$) δ 6.98 (1H, s, H1), 3.72 (3H, s, H21), 3.65 (3H, s, H17), 2.24–1.14 (20H, m, H6, 7', 7'', 8', 8'', 9', 9'', H10, 11', 11'', 12', 12'', 13', 13'', 14', 14'', 15', 15'').

Figure S22. ^{1}H NMR spectrum of **6**.

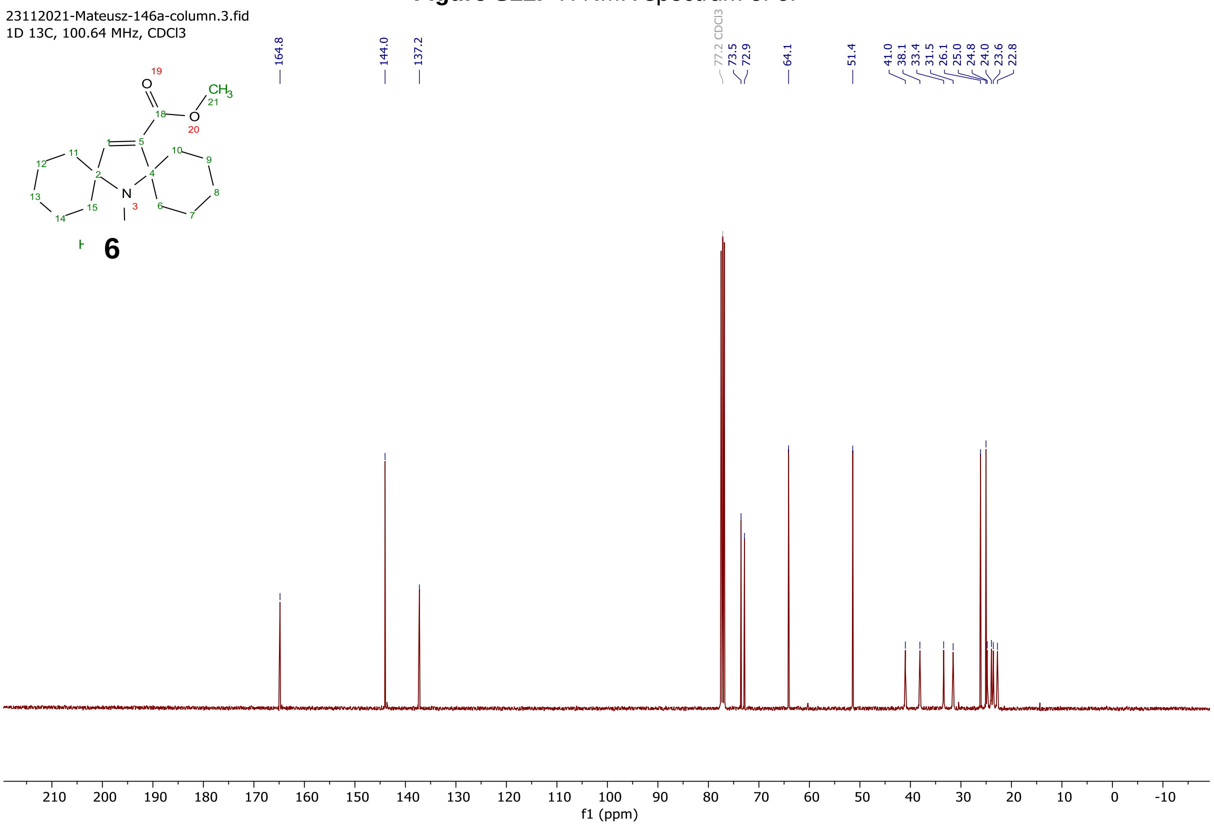

^{13}C NMR (101 MHz, $CDCl_3$) δ 164.8, 144.0, 137.2, 73.5, 72.9, 64.1, 51.4, 41.0, 38.1, 33.4, 31.5, 26.1, 25.0, 24.8, 24.0, 23.6, 22.8.

Figure S23. ^{13}C NMR spectrum of **6**.

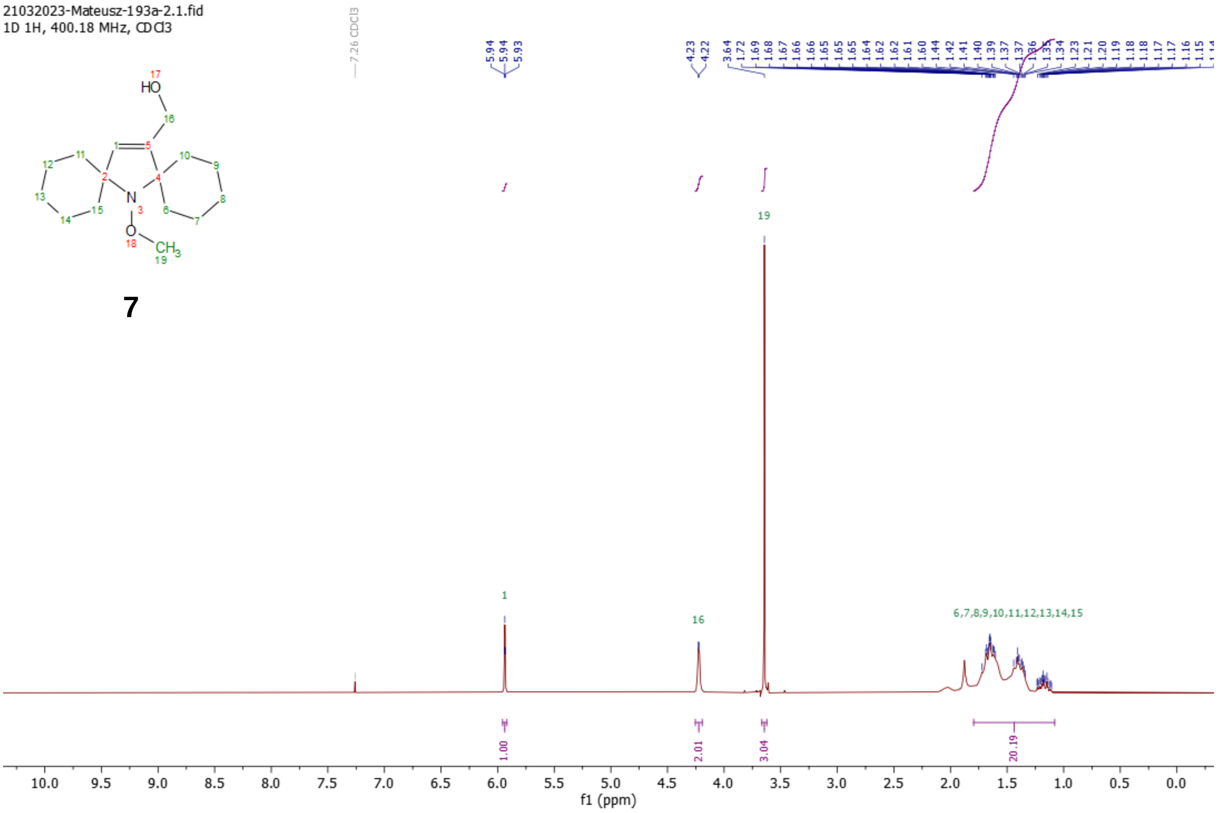

^{1}H NMR (400 MHz, $CDCl_3$) δ 5.94 (1H, t, J = 1.8 Hz, H1), 4.22 (2H, d, J = 4.3 Hz, H16), 3.64 (3H, s, H19), 1.80–1.08 (20H, m, H6, H7, H8, H9, H10, H11, H12, H13, H14, H15).

Figure S24. ^{1}H NMR spectrum of **7**.

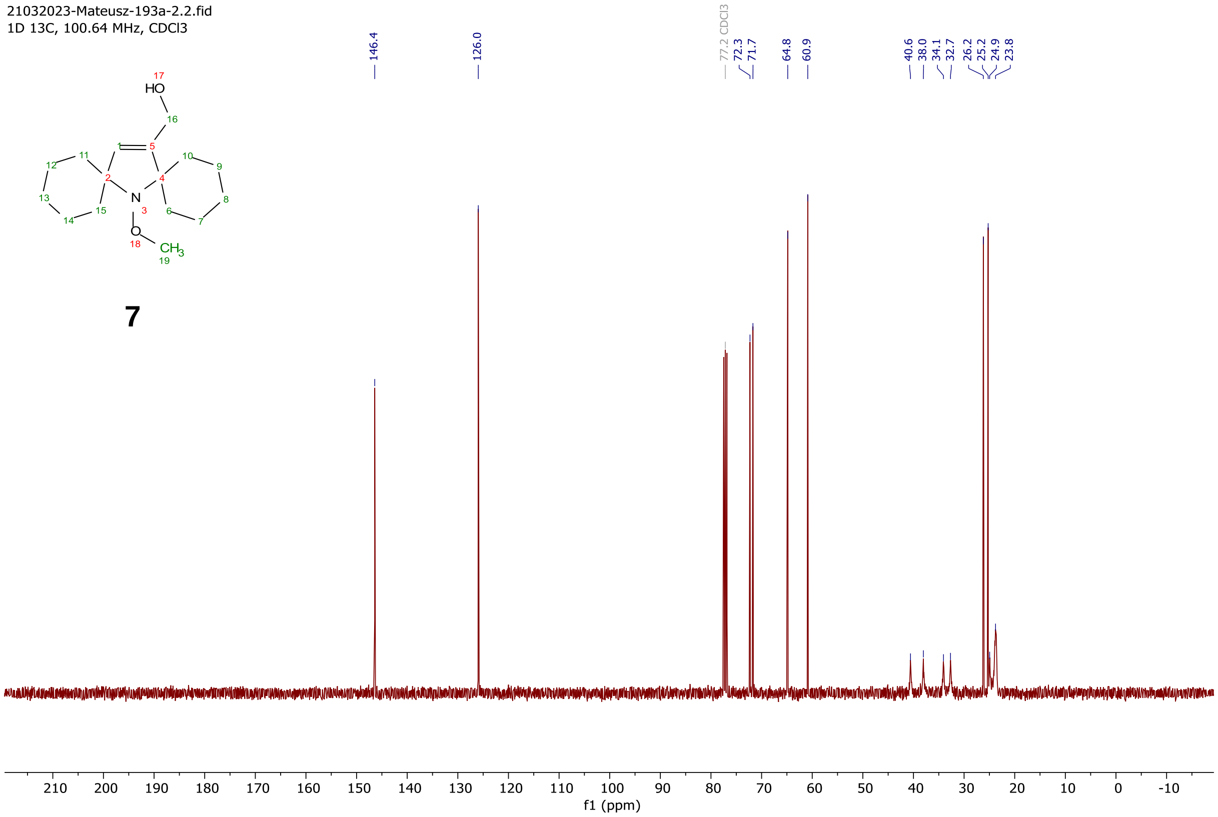

^{13}C NMR (101 MHz, $CDCl_3$) δ 146.4, 126.0, 72.3, 71.7, 64.8, 60.9, 40.6, 38.0, 34.1, 32.7, 26.2, 25.2, 24.9, 23.8.

Figure S25. ^{13}C NMR spectrum of **7**.

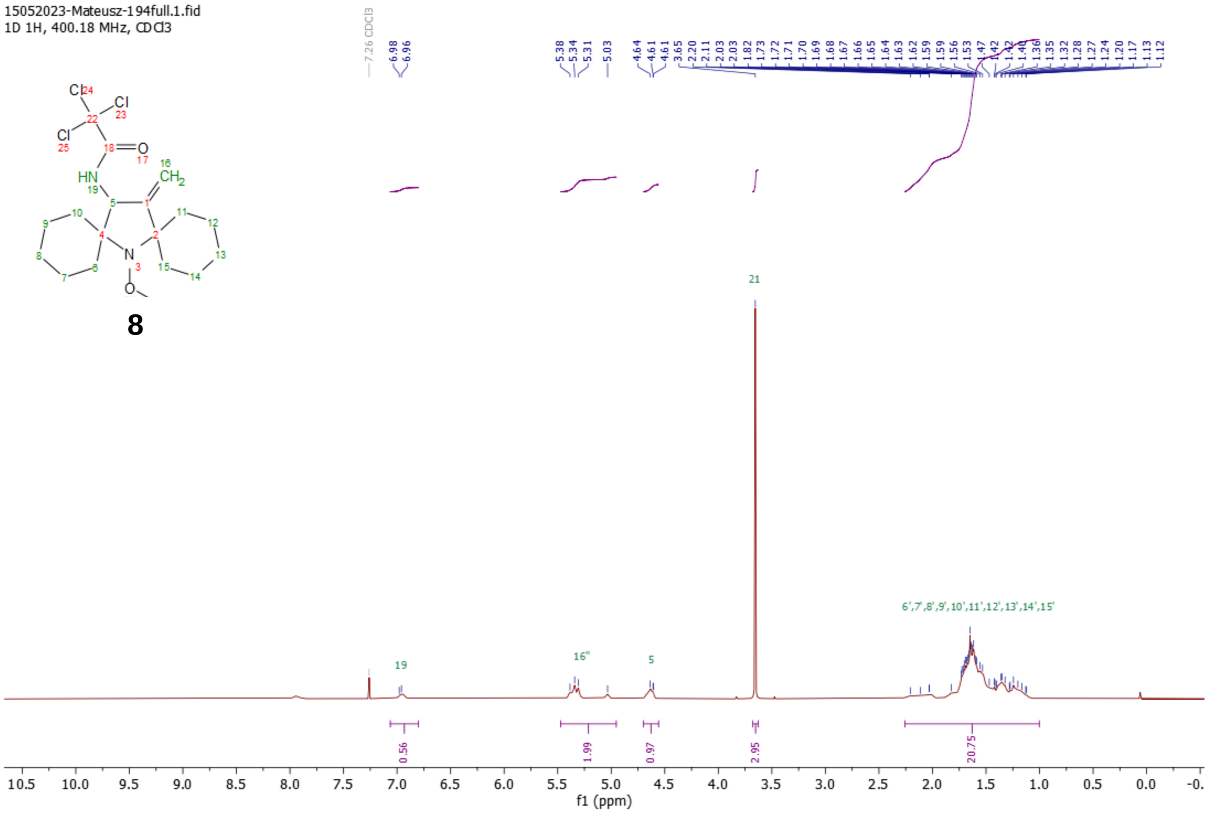

^{1}**H NMR** (400 MHz, $CDCl_3$) δ 6.96 (1H, s, H19), 5.47–4.95 (2H, m, 16''), 4.70–4.56 (1H, m, H5), 3.65 (3H, s, H21), 2.49–1.00 (20H, m, 6', 7', 8', 9', 10', 11', 12', 13', 14', 15').

Figure S26. ^{1}H NMR spectrum of **8**.

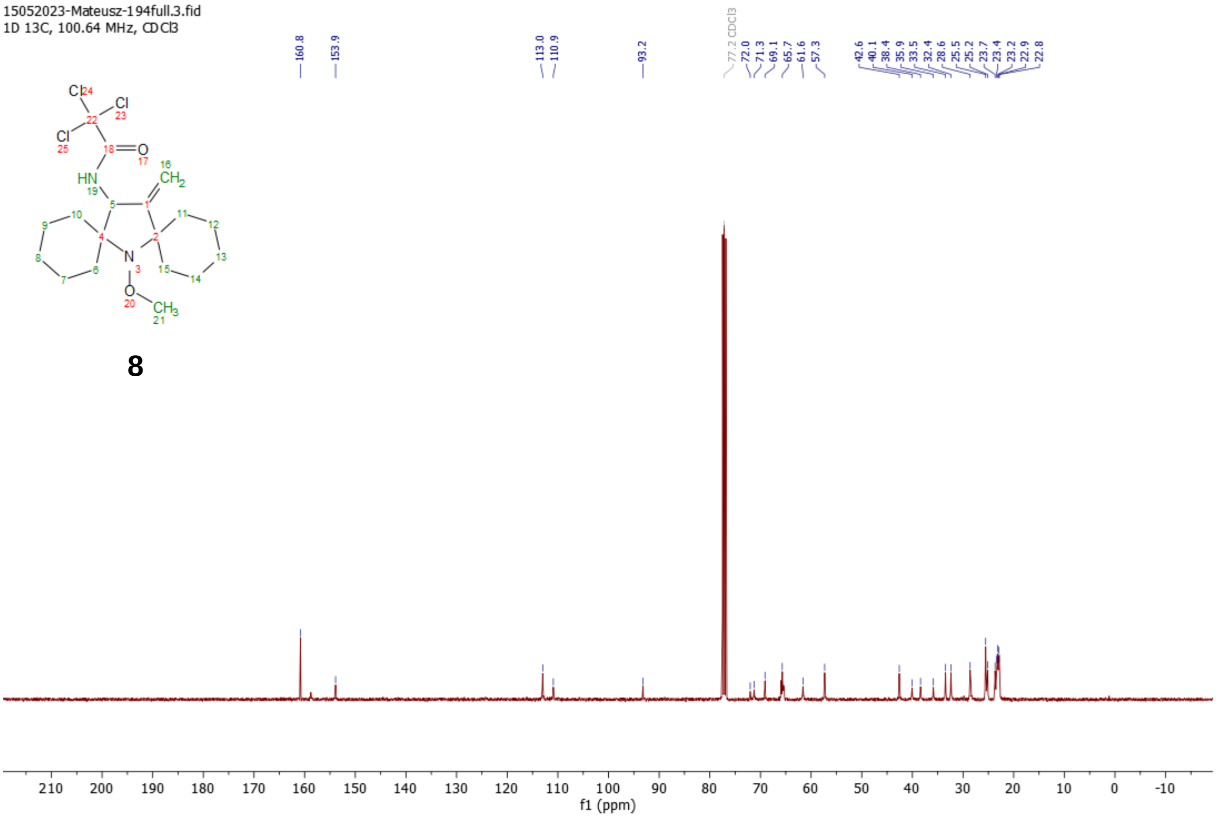

^{13}C NMR (101 MHz, $CDCl_3$) δ 160.8, 153.9, 113.0, 110.9, 93.2, 72.0, 71.3, 69.1, 65.7, 61.6, 57.3, 42.6, 40.1, 38.4, 35.9, 33.5, 32.4, 28.6, 25.5, 25.2, 23.7, 23.4, 23.2, 22.9, 22.8.

Figure S27. ^{13}C NMR spectrum of **8**.

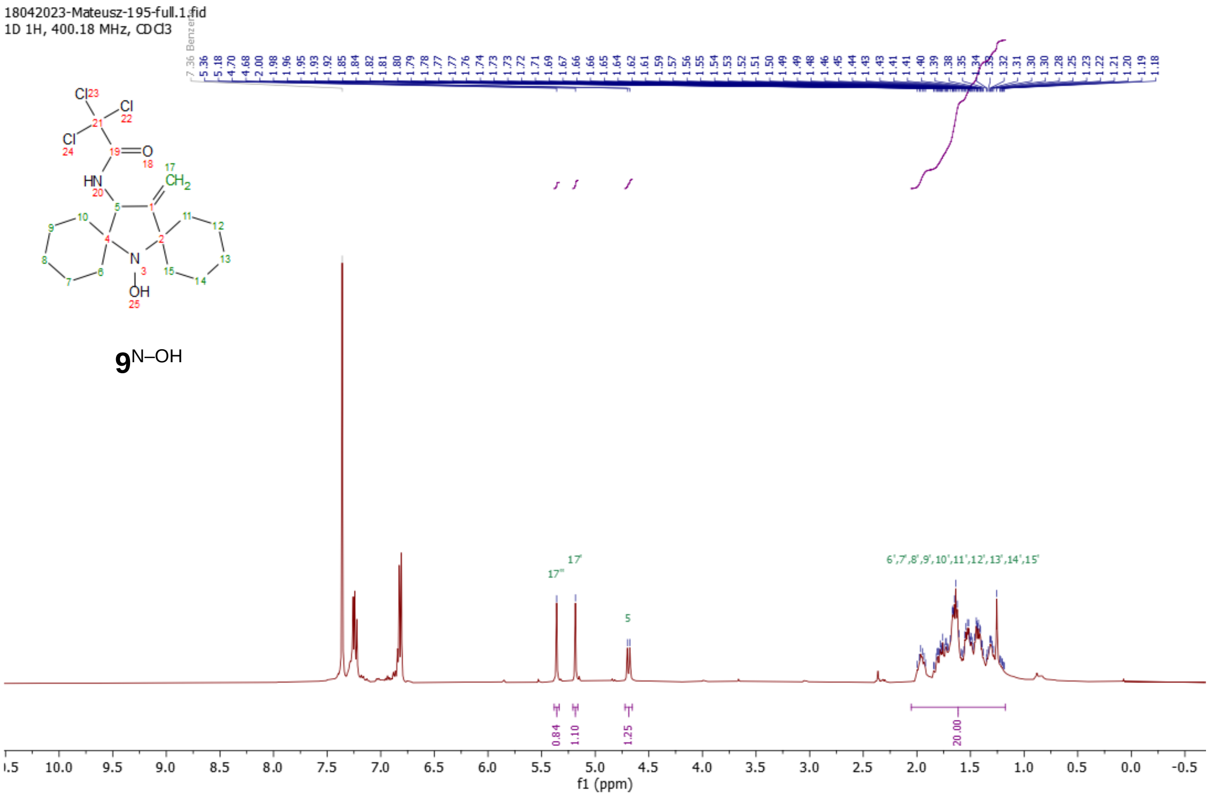

^{1}H NMR (400 MHz, $CDCl_3$) δ 5.36 (1H, s, 17''), 5.18 (1H, s, 17'), 4.69 (1H, d, J = 9.4 Hz, H5), 2.05–1.18 (20H, m, 6', 7', 8', 9', 10', 11', 12', 13', 14', 15').

Figure S28. ^{1}H NMR spectrum of **9** after addition of phenylhydrazine.

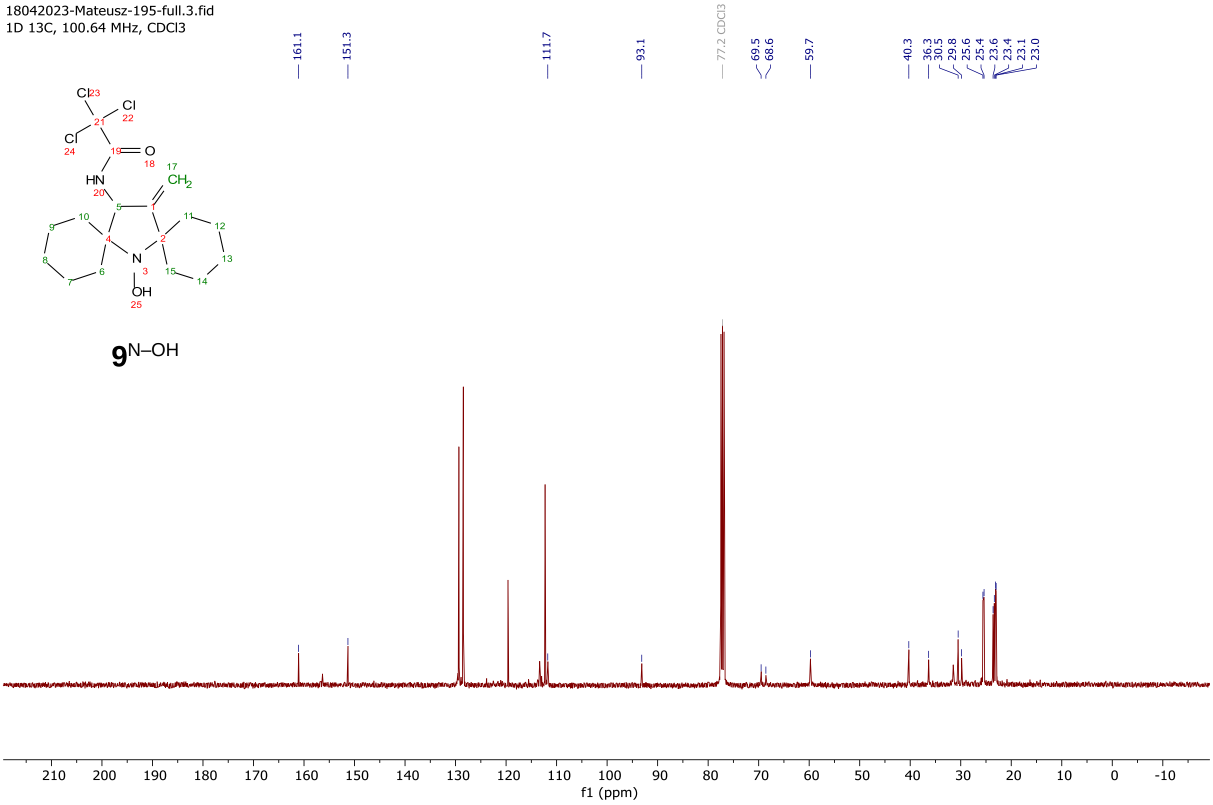

^{13}C NMR (101 MHz, $CDCl_3$) δ 161.1, 151.3, 111.7, 93.1, 69.5, 68.6, 59.7, 40.3, 36.3, 30.5, 29.8, 25.6, 25.4, 23.6, 23.4, 23.1, 23.0.

Figure S29. ^{13}C NMR spectrum of **9** after addition of phenylhydrazine.

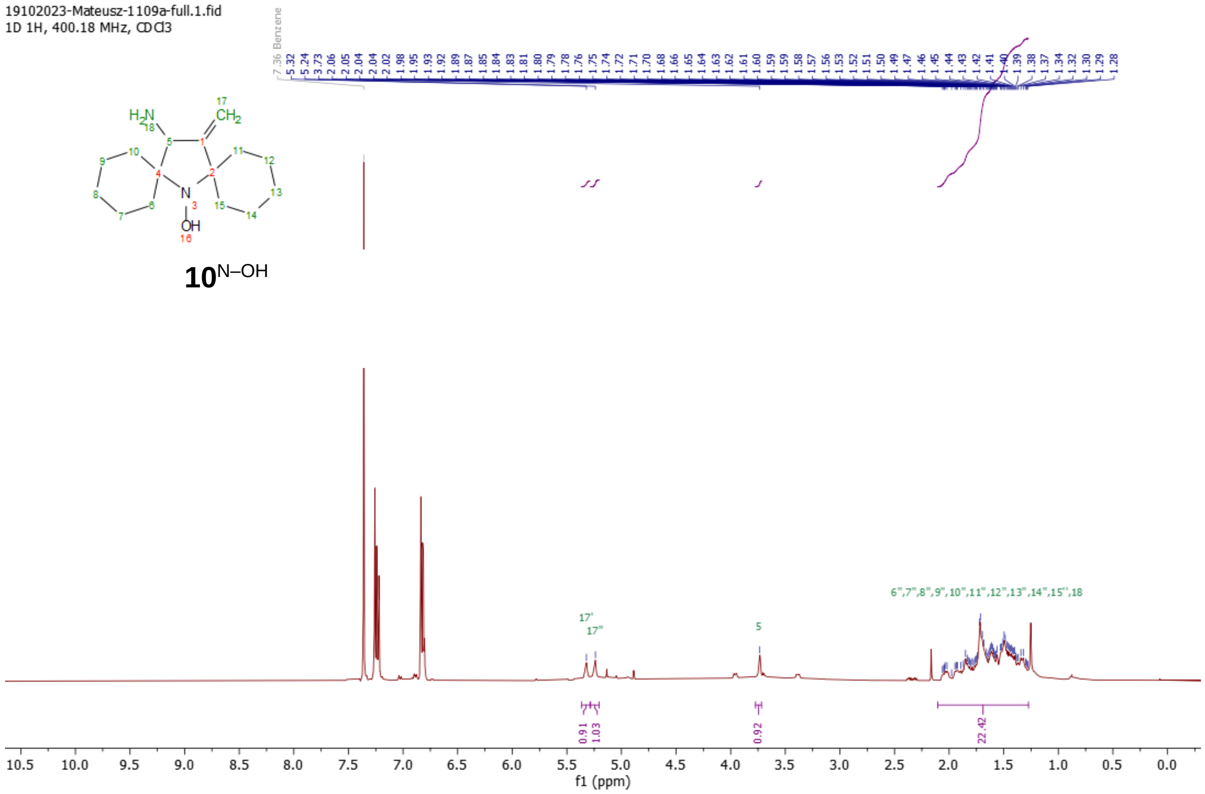

^{1}**H NMR** (400 MHz, $CDCl_3$) δ 5.32 (1H, s, 17'), 5.24 (1H, s, 17''), 3.73 (1H, s, H5), 2.11–1.27 (20H, m, 6'', 7'', 8'', 9'', 10'', 11'', 12'', 13'', 14'', 15'').

Figure S30. ^{1}H NMR spectrum of **10** after addition of phenylhydrazine.

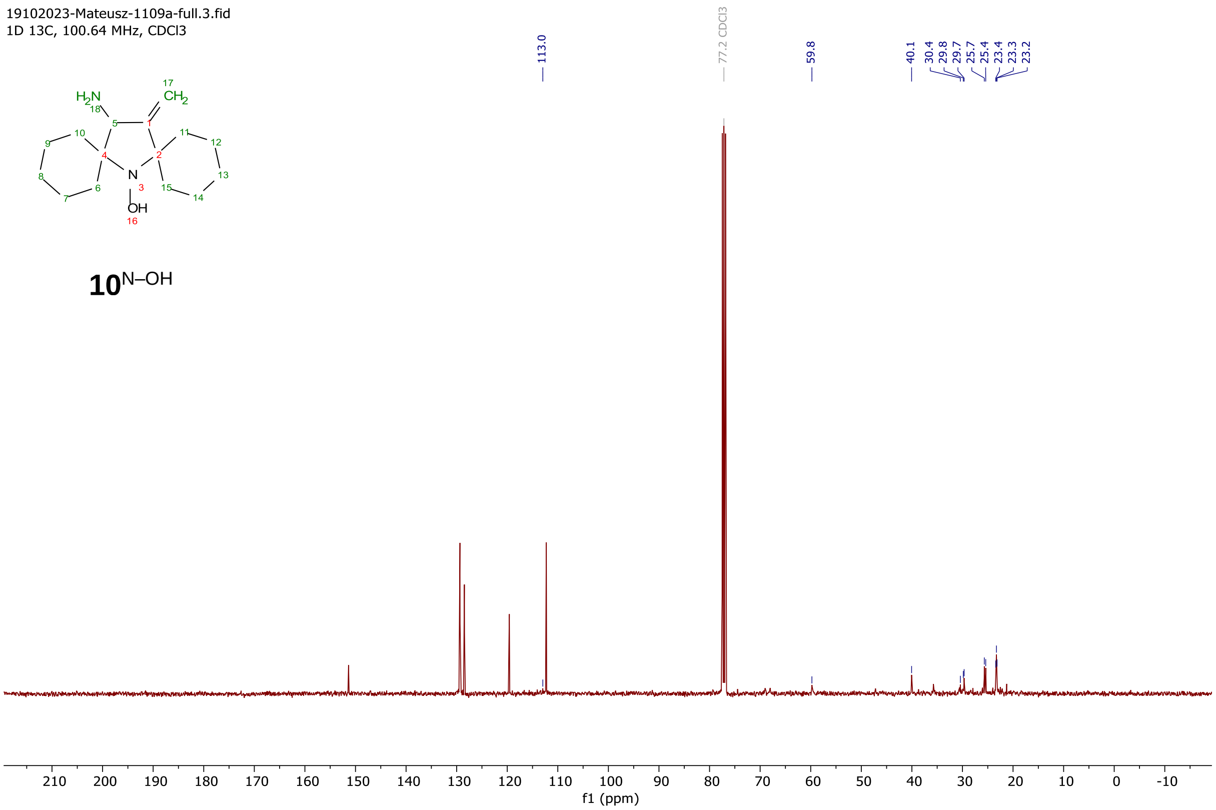

^{13}C NMR (101 MHz, $CDCl_3$) δ 113.0, 59.8, 40.1, 30.4, 29.8, 29.7, 25.7, 25.4, 23.4, 23.3, 23.2.

Figure S31. ^{13}C NMR spectrum of **10** after addition of phenylhydrazine.

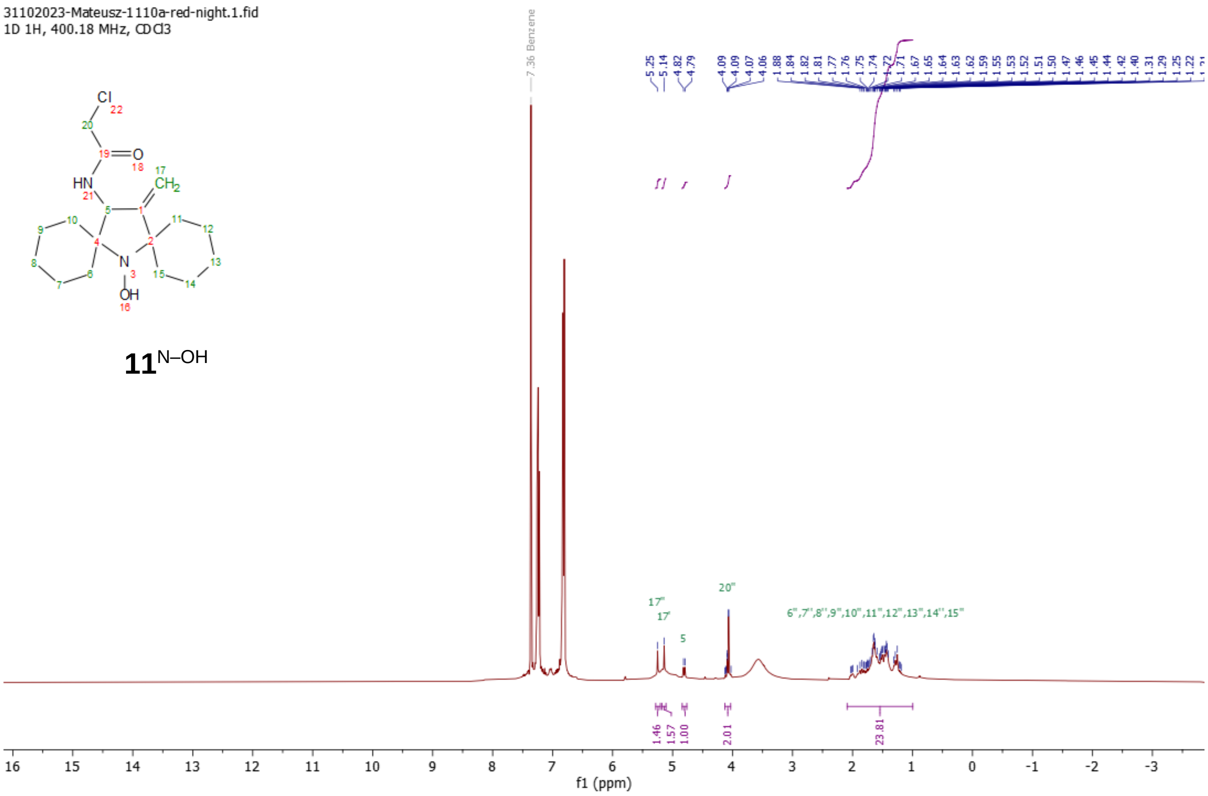

^{1}**H NMR** (400 MHz, $CDCl_3$) δ 5.25 (1H, s, 17''), 5.14 (1H, s, 17'), 4.81 (1H, d, *J* = 9.8 Hz, H5), 4.13–4.03 (2H, m, 20''), 2.09–1.00 (20H, m, 6'', 7'', 8'', 9'', 10'', 11'', 12'', 13'', 14'', 15'').

Figure S32. ^{1}H NMR spectrum of **11** after addition of phenylhydrazine.

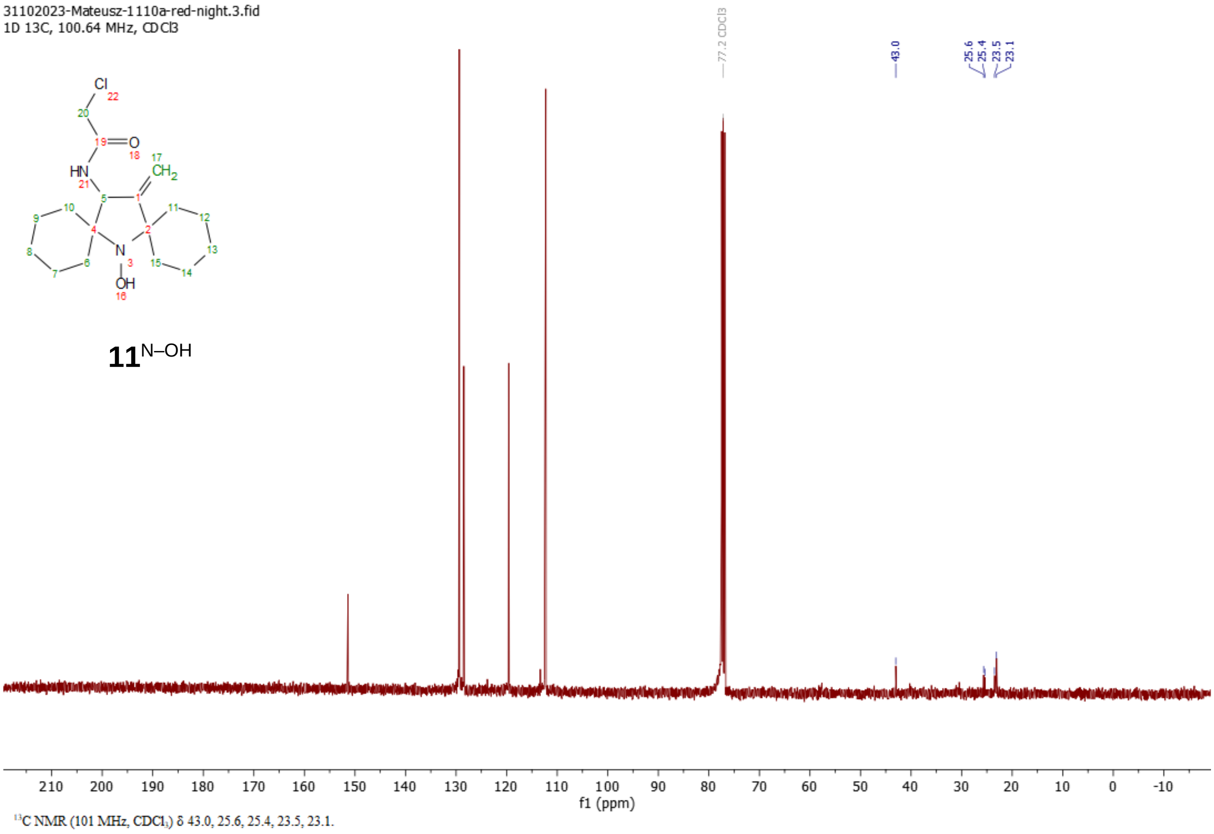

^{13}C NMR (101 MHz, $CDCl_3$) δ 43.0, 25.6, 25.4, 23.5, 23.1.

Figure S33. ^{13}C NMR spectrum of **11** after addition of phenylhydrazine.

References

1. S. Morisako, R. Shang and Y. Yamamoto, *Inorg. Chem.*, 2016, **55**, 10767-10773.
2. Y. Wang, J. T. Paletta, K. Berg, E. Reinhart, S. Rajca and A. Rajca, *Org. Lett.*, 2014, **16**, 5298-5300.
3. B. A. Chalmers, J. C. Morris, K. E. Fairfull-Smith, R. S. Grainger and S. E. Bottle, *Chem. Commun.*, 2013, **49**, 10382-10384.
4. H. Yamanaka, K. Sato, H. Sato, M. Iida, T. Oishi and N. Chida, *Tetrahedron*, 2009, **65**, 9188-9201.
5. S. Bleicken, T. E. Assafa, H. Zhang, C. Elsner, I. Ritsch, M. Pink, S. Rajca, G. Jeschke, A. Rajca and E. Bordignon, *ChemistryOpen*, 2019, **8**, 1057-1065.
6. CrysAlisPro, version, Rigaku Oxford Diffraction, Rigaku Corporation, Tokyo, Japan, 2023.
7. G. M. Sheldrick, *Acta Crystallogr. Sect. A Found. Adv.*, 2015, **71**, 3-8.
8. G. M. Sheldrick, *Acta Crystallogr. Sect. C Struct. Chem.*, 2015, **71**, 3-8.
9. O. V. Dolomanov, L. J. Bourhis, R. J. Gildea, J. A. K. Howard and H. Puschmann, *J. Appl. Cryst.*, 2009, **42**, 339-341.
10. E. M. Mocanu, Y. Ben-Ishay, L. Topping, S. R. Fisher, R. I. Hunter, X.-C. Su, S. J. Butler, G. M. Smith, D. Goldfarb and J. E. Lovett, *Appl. Magn. Reson.*, 2025, DOI: 10.1007/s00723-024-01741-0.
11. The PyMOL Molecular Graphics System, version, Schrödinger, LLC, 2015.
12. M. Ikura, G. M. Clore, A. M. Gronenborn, G. Zhu, C. B. Klee and A. Bax, *Science*, 1992, **256**, 632-638.
13. M. Pannier, S. Veit, A. Godt, G. Jeschke and H. W. Spiess, *J. Magn. Reson.*, 2000, **142**, 331-340.
14. A. D. Milov, A. B. Ponomarev and Y. D. Tsvetkov, *Chem. Phys. Lett.*, 1984, **110**, 67-72.
15. A. D. Milov, K. M. Salikhov and M. D. Shirov, *Fiz. Tverd. Tela*, 1981, **23**, 975-982.
16. O. Schiemann, C. A. Heubach, D. Abdullin, K. Ackermann, M. Azarkh, E. G. Bagryanskaya, M. Drescher, B. Endeward, J. H. Freed, L. Galazzo, D. Goldfarb, T. Hett, L. Esteban Hofer, L. Fábregas Ibáñez, E. J. Hustedt, S. Kucher, I. Kuprov, J. E. Lovett, A. Meyer, S. Ruthstein, S. Saxena, S. Stoll, C. R. Timmel, M. Di Valentin, H. S. McHaourab, T. F. Prisner, B. E. Bode, E. Bordignon, M. Bennati and G. Jeschke, *J. Am. Chem. Soc.*, 2021, **143**, 17875-17890.
17. S. G. Worswick, J. A. Spencer, G. Jeschke and I. Kuprov, *Sci. Adv.*, 2018, **4**, eaat5218.
18. J. Keeley, T. Choudhury, L. Galazzo, E. Bordignon, A. Feintuch, D. Goldfarb, H. Russell, M. J. Taylor, J. E. Lovett, A. Eggeling, L. Fábregas Ibáñez, K. Keller, M. Yulikov, G. Jeschke and I. Kuprov, *J. Magn. Reson.*, 2022, **338**, 107186.
19. G. Jeschke, V. Chechik, P. Ionita, A. Godt, H. Zimmermann, J. Banham, C. R. Timmel, D. Hilger and H. Jung, *Appl. Magn. Reson.*, 2006, **30**, 473-498.
20. M. H. Tessmer and S. Stoll, *PLoS Comput. Biol.*, 2023, **19**, e1010834.
